# Inferring and perturbing cell fate regulomes in human brain organoids

**DOI:** 10.1038/s41586-022-05279-8

**Published:** 2022-10-05

**Authors:** Jonas Simon Fleck, Sophie Martina Johanna Jansen, Damian Wollny, Fides Zenk, Makiko Seimiya, Akanksha Jain, Ryoko Okamoto, Malgorzata Santel, Zhisong He, J. Gray Camp, Barbara Treutlein

**Affiliations:** 1 Department of Biosystems Science and Engineering, ETH Zürich, Basel, Switzerland; 2 Max Planck Institute for Evolutionary Anthropology, Leipzig, Germany; 3 Institute of Molecular and Clinical Ophthalmology Basel, Basel, Switzerland; 4 University of Basel, Basel, Switzerland; 5 Present Address: Roche Institute for Translational Bioengineering (ITB), Roche Pharma Research and Early Development, Roche Innovation Center Basel, Basel, Switzerland

**Keywords:** Neurogenesis, Gene expression, Stem-cell differentiation, Regulatory networks, RNA sequencing

## Abstract

Self-organizing neural organoids grown from pluripotent stem cells^1–3^ combined with single-cell genomic technologies provide opportunities to examine gene regulatory networks underlying human brain development. Here we acquire single-cell transcriptome and accessible chromatin data over a dense time course in human organoids covering neuroepithelial formation, patterning, brain regionalization and neurogenesis, and identify temporally dynamic and brain-region-specific regulatory regions. We developed Pando—a flexible framework that incorporates multi-omic data and predictions of transcription-factor-binding sites to infer a global gene regulatory network describing organoid development. We use pooled genetic perturbation with single-cell transcriptome readout to assess transcription factor requirement for cell fate and state regulation in organoids. We find that certain factors regulate the abundance of cell fates, whereas other factors affect neuronal cell states after differentiation. We show that the transcription factor GLI3 is required for cortical fate establishment in humans, recapitulating previous research performed in mammalian model systems. We measure transcriptome and chromatin accessibility in normal or GLI3-perturbed cells and identify two distinct GLI3 regulomes that are central to telencephalic fate decisions: one regulating dorsoventral patterning with HES4/5 as direct GLI3 targets, and one controlling ganglionic eminence diversification later in development. Together, we provide a framework for how human model systems and single-cell technologies can be leveraged to reconstruct human developmental biology.

## Abstract (web summary)

A multi-omic atlas of brain organoid development facilitates the inference of an underlying gene regulatory network using the newly developed Pando framework and shows—in conjunction with perturbation experiments—that GLI3 controls forebrain fate establishment through interaction with HES4/5 regulomes.

## Main

The ability to generate complex brain-like tissue in controlled culture environments from human stem cells offers great promise to understand the mechanisms that underlie human brain development. Cerebral or other unguided neural organoids develop from embryonic stem (ES) cells or induced pluripotent stem (iPS) cells into a three-dimensional neuroepithelium that self-patterns, regionalizes and, ultimately, forms neurons of the different brain regions^1–3^. The fate and state of each cell is orchestrated in part through complex circuits of transcription factors (TFs), converging at regulatory elements and interacting with chromatin to enable precise control of gene expression. Single-cell sequencing approaches enable the profiling of gene expression and chromatin accessibility in individual cells, which opens up new opportunities to survey the set of regulatory control features in any given cell type or state (regulomes). Comprehensive mouse and human brain cell atlases can be used as a reference for understanding organoid cell composition and development^4–6^. Direct comparisons between organoids and primary counterparts in mouse and human have quantified a notable similarity between the neural progenitor and neuronal transcriptome profiles^7–9^. Brain organoids have been used to successfully model microcephaly^2^, periventricular heterotopia^10^, autism^11^ and other neurodevelopmental disorders^12,13^ that may have differential effects on the various human brain regions. However, we do not yet understand the gene regulatory networks (GRNs) that coordinate early human brain development in normal and perturbed conditions.

Research in model systems has identified core signalling factors and gene regulatory programs that orchestrate brain region formation in vertebrates. Initially, extrinsic signals establish an anterior–posterior axis that triggers additional localized gradients downstream to segment the neural tube into distinct brain regions. Combinatorial activities of morphogens, including SHH, WNTs, BMPs, FGFs, NOTCH, neuregulins and R-spondins, converge on transcription factors to execute regionalization. Much of what is known about these pathways in regulating brain morphogenesis has been examined in non-human model systems, and it remains unclear how human brain development has diverged from our mammalian ancestors. Moreover, detailed studies of the mechanisms controlling multiregion brain organoids may provide new insights into the process of brain self-organization^14^.

New single-cell genomic methods enable high-throughput and quantitative analysis of single-cell transcriptomes and accessible chromatin profiles. These features can also be quantified within an individual cell in a multi-omic measurement, providing insights into gene expression and regulation in the same cell. Furthermore, CRISPR–Cas gene editing coupled with single-cell transcriptome readouts^15–17^ enables pooled genetic perturbation experiments in vivo^18^. These strategies and vector systems, combined with functionalization of human iPS cells with inducible CRISPR–Cas9 systems, provide an opportunity to perturb gene function in brain organoids, and systematically assess the effects across human brain regions.

Here we used a multimodal approach to examine cell-fate regulation during human early brain development. We first built a regulome from single-cell transcriptome and accessible chromatin profiling data across a brain organoid developmental time course. Regulome perturbations using multiplexed CRISPR perturbation experiments in organoids identified effects on regional fate decisions as well as effects on cell states after fate acquisition. Multiome analysis of a critical period of brain region formation in *GLI3-*knockout (KO) and Sonic Hedgehog signalling molecule (SHH)-exposed organoids revealed regulatory disruption of dorsoventral telencephalon diversification and, with the help of the inferred regulome, we distinguished direct and indirect targets of GLI3. Together, we established a regulome perspective to understand and examine early human brain development.

## Multi-omic view of organoid development

**Fig. 1:**
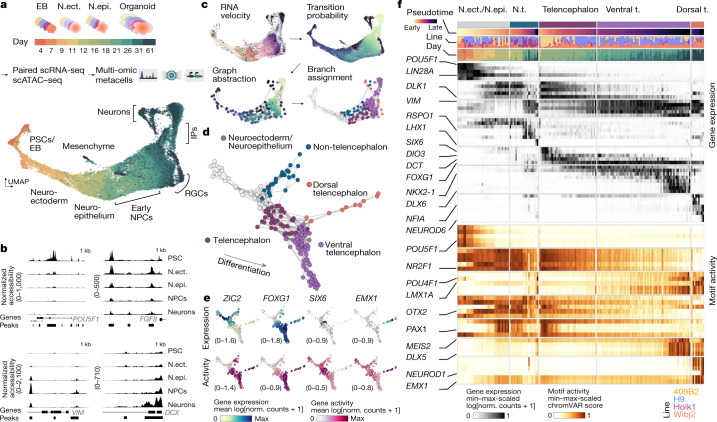
Multi-omic atlas of brain organoid development reveals developmental hierarchies and critical stages of fate decision. **a**, Schematic of the experimental design and UMAP embedding of integrated multi-omic metacells. Organoids from three iPS cell lines and one ES cell line were dissociated for paired scRNA-seq and scATAC-seq at time points spanning 4 days to 2 months of development. The two modalities were integrated to form metacells with RNA and ATAC components. EB, embryoid body; IPs, intermediate progenitors; N.ect., neuroectoderm; N.epi., neuroepithelium; PSCs, pluripotent stem cells. **b**, Examples of loci with differential accessibility during organoid development from pluripotency. **c**, Schematic of the branch-inference strategy. High-resolution clusters were assigned to branches on the basis of terminal fate transition probabilities calculated based on RNA velocity. **d**, Branch visualization in a force-directed layout. The circles represent high-resolution clusters of metacells coloured by assignment (neuroepithelium (grey); non-telencephalon progenitors (teal); telencephalon progenitors (plum); dorsal telencephalon (orange); ventral telencephalon (purple)). **e**, Graph representation of regional branches coloured by mean expression (log[transcript counts per 10,000 + 1]) (top) and gene activity (log[transcript counts per 10,000 + 1]) (bottom) of marker genes. The range of values is indicated for each plot. Norm., normalized. **f**, Stage- and branch-specific gene expression and motif enrichment *z*-score (Methods). Values are minimum–maximum (min–max) scaled across rows. N.t., non-telencephalon; t., telencephalon.

**Extended Data Fig. 1:**
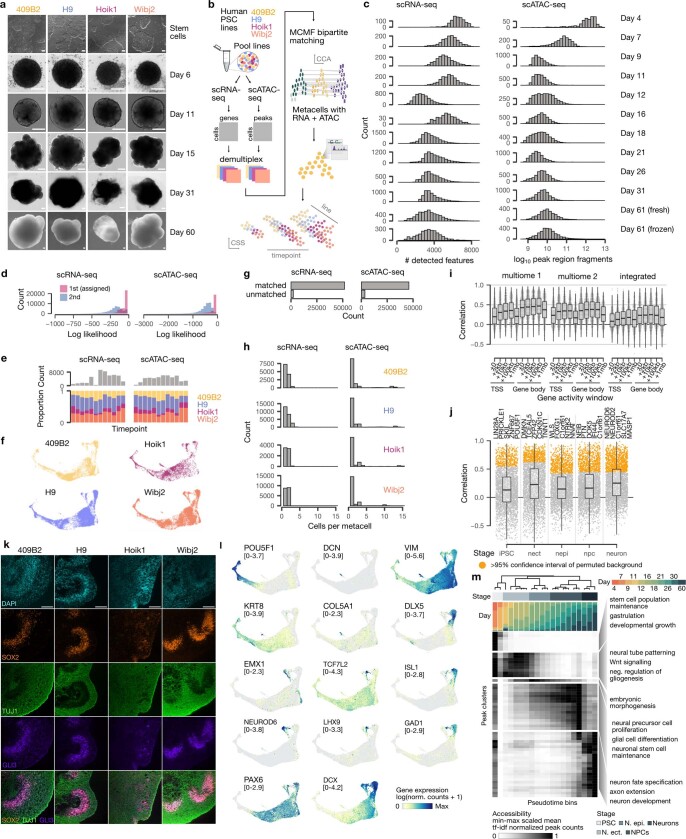
Supplemental analysis of brain organoid developmental multiome data. a, Phase contrast (until day 15) and bright field (day 31–60) showing examples of different stages of organoid development for four different stem cell lines. Images are representative for 96 organoids per line. Scale bar is 200 µm. b, Schematic of the experimental design and data integration strategy. c, Histogram of scRNA-seq and scATAC-seq quality control metrics. d, Histograms showing assignment log likelihoods for demultiplexing based on single nucleotide variants. e, Bar plot of number of cells for each time point (top) and stacked barplot showing proportion of cell lines (bottom) at different time points. f, Distribution of iPS cell (iPSC) lines on the UMAP embedding. g, Bar plots showing number matched and unmatched cells during MCMF bipartite matching. h, Histogram showing the number of cells per metacell for each cell line. i, Box plots showing correlation between gene expression and gene activity metrics for two multiome experiments and the integrated metacells (n = 477 genes). j, Box plots showing correlation split by stage (n = 3527 genes). Genes >95% confidence intervals of correlation to permuted background are coloured in yellow. Box center represents the median, boxes indicate 25%–75% interquantile range and whiskers 1.5 * interquantile range. k, Immunohistochemical staining for progenitor cells (SOX2, orange and GLI3, purple) and neurons (TUJ1, green) for 2 month old organoids of four cell lines. DAPI is shown in cyan. Scale bar: 200 μm. l, UMAP embedding coloured by marker gene expression (log(transcript counts per 10k+1)). The range of values is indicated for each plot. m, Hierarchical clustering of pseudotemporal bins. Top bars show stage and proportion of time points per bin. Heatmap shows min-max scaled mean accessibility (tf-idf normalized fragment counts) of stage-specific peak clusters for each pseudotime bin. Representative GREAT enrichments are shown for each stage.

To examine the mechanisms that underlie human brain development, we generated single-cell transcriptome and single-cell accessible chromatin profiling data over a time course of brain organoid development (Fig. 1a, Extended Data Fig. 1a and Supplementary Table 1). The dataset incorporates 11 time points from 3 human iPS cell lines and 1 ES cell line covering 2 months of development spanning embryoid body formation, neuroectoderm induction, neuroepithelialization, neural progenitor patterning and neurogenesis. At each time point, organoid tissues from the four lines were dissociated and single-cell RNA-sequencing (scRNA-seq) and single-cell assay for transposase-accessible chromatin with sequencing (scATAC–seq) pipelines (10x Genomics) were run on the same cell suspension. The sequencing data were demultiplexed using single-nucleotide variants specific to each individual and the two modalities for each line and time point were integrated using canonical correlation analysis (CCA)^19^ (Extended Data Fig. 1b–f and Supplementary Table 2). We constructed ‘multi-omic metacells’ containing information on both transcriptome and chromatin accessibility using minimum-cost, maximum-flow bipartite matching^20^ within the CCA space (Extended Data Fig. 1b,g,h). We evaluated the integration using a multiome dataset, in which the transcriptome and accessible chromatin were measured within the same cell, and observed strong correlation (Extended Data Fig. 1i,j). The metacells were integrated using cluster similarity spectrum (CSS)^21^, and the integrated data were visualized using uniform manifold approximation and projection (UMAP) embedding. This revealed a relatively continuous distribution of cell states through the entire time course (Fig. 1a). Organoid development proceeds from pluripotency (for example, *POU5F1*) through a neural progenitor cell (NPC) state (for example, *PAX6*, *VIM*) to progenitor and neuron cell states of the dorsal telencephalon (for example, *EMX1*, *NEUROD6*), the ventral telencephalon (for example, *DLX5*, *ISL1*, *GAD1*), of non-telencephalic regions (for example, *TCF7L2*, *LHX9*) and of a small mesenchymal population (for example, *DCN*, *COL5A1*), with cells from the different lines largely intermixed (Extended Data Fig. 1f,k,l). The high-dimensionality of the data could be used to identify marker genes and gene regulatory regions for the different cell states (Fig. 1b, Extended Data Fig. 1l and Supplementary Table 3). We observed a pseudotemporal cascade of chromatin accessibility changes over the developmental time course associated with genes involved in stem cell maintenance, neural tube patterning, morphogenesis, neural precursor proliferation, neuron fate specification and other relevant biological processes (Extended Data Fig. 1m and Supplementary Table 4).

**Extended Data Fig. 2:**
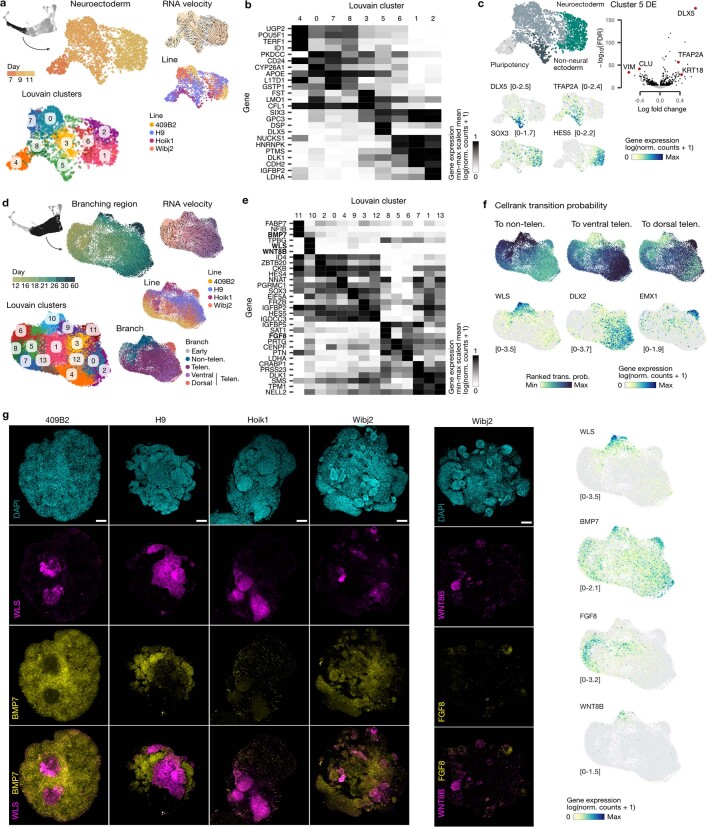
Heterogeneity analysis in different stages of organoid development. a,d, UMAP embedding of a subset of the organoid trajectory surrounding neuroectoderm cells (a) and the branching window (d) coloured by time point, velocity pseudotime, cell line, branch prediction and lovain clusters. b,e, Heatmap showing mean min-max scaled expression (log(transcript counts per 10k + 1)) of cluster markers. c, UMAP embedding coloured by cluster identities, expression patterns of cluster markers. Volcano plot shows differentially expressed (DE) genes of cluster 5 relative to other clusters. f, UMAP embedding coloured by rank-transformed CellRank transition probability to non-telencephalon, ventral telencephalon and dorsal telencephalon and coloured by expression of selected transcription factors. g, Whole-mount HCR in situ hybridizations of day 18 organoids and UMAP embedding coloured by expression of targets. Stainings were performed on 2-3 organoids per cell line and representative images were shown. Scale bar: 100 μm. The range of expression values is indicated for each feature plot.

Previous studies have described the emergence of patterning centres within the neuroepithelium that coordinate to regionalize the developing organoid^22^. To reconstruct the earliest events involved in cell-fate restriction, we subclustered early portions of the trajectory and identified molecular heterogeneity (Extended Data Fig. 2). In the initial stages (day 7–9), we observed a predominant neuroectodermal population (*SIX3*, *CDH2*, *SOX3*, *HES5*) and a minor population of cells expressing non-neural ectoderm markers (*DLX5*, *TFAP2A*)^23,24^ (Extended Data Fig. 2a–c). After day 9, cells differentiate into a neuroepithelial population (*LDHA*), which later diverges into NPCs expressing either telencephalic (*FGF8*) and non-telencephalic markers (*WLS*, *WNT8B*), followed by a second divergence into dorsal (*BMP7*, *EMX1*) and ventral telencephalic NPCs (*DLX2*; Extended Data Fig. 2d–f). RNA fluorescence in situ hybridization (RNA-FISH) using hybridized chain reactions (HCR) of whole-mount 18-day-old organoids confirmed the expression and spatial segregation of some of these regional markers (Extended Data Fig. 2g).

**Extended Data Fig. 3:**
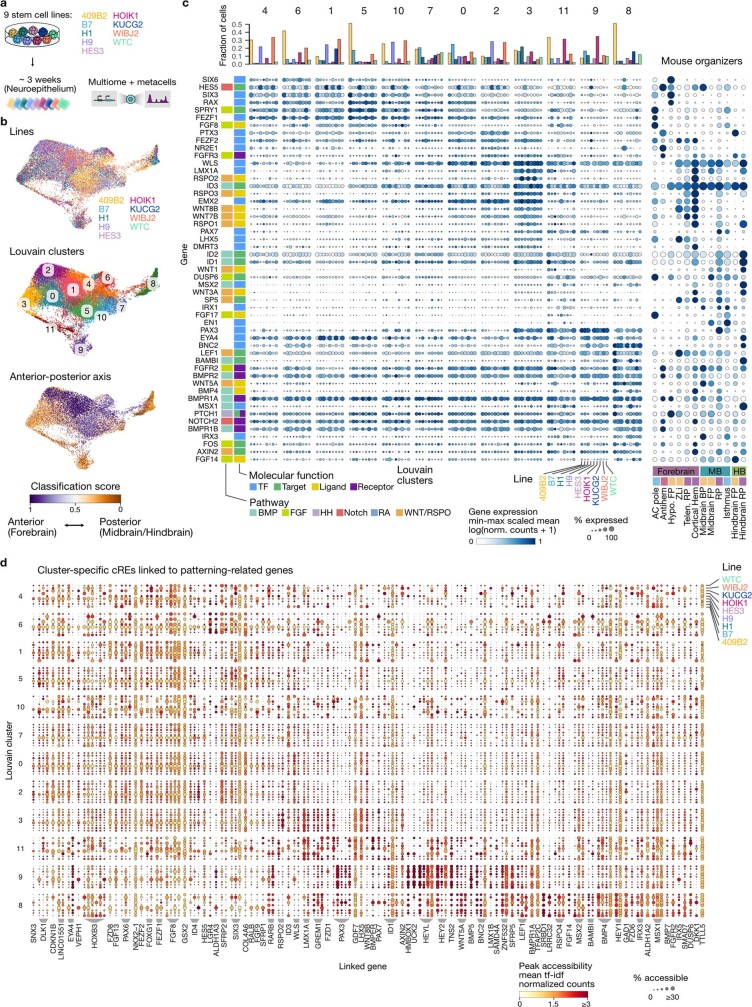
Signalling transcriptome and regulatory element landscape of the organoid neuroepithelium from 9 stem cell lines. a, Schematic of the experimental setup. Multiome quantification was performed on organoids in the neuroepithelial stage (~3 weeks) from a total of 9 stem cell lines. The data was combined with the data from the same stage in the early time course. b, UMAP embedding coloured by cell line, louvain clusters and anterior-posterior axis (forebrain versus non-forebrain) classification score. c, Bar plots (top) showing fraction of cells per cell line in each cluster. Dotplot (left) showing min-max scaled expression (log(transcript counts per 10k + 1)) (colour) and proportion of expressing cells (dot size) for transcription factors (TFs) and genes from different signalling pathways in clusters of 3 week old organoid data set split by cell line. All genes are annotated as TF, receptor, ligand, or TF target and if applicable, coloured by the related signalling pathway. Dotplot (right) showing expression (colour) and proportion of expressing cells (dot size) for the same genes of Extended Data Fig. 3d in mouse developing brain organizer cells of different brain regions^6^. d, Dot plot showing cluster-specific cis regulatory elements (CREs) linked to patterning genes split by different cell lines. Colour and size indicate peak accessibility (if-idf normalized fragment counts) and proportion of expressing cells, respectively.

To assess the neuroepithelial self-patterning variation across stem cell lines, we collected additional single-cell multiome data including transcriptome and accessible chromatin modalities for a total of 9 lines (iPS cells: 409B2, B7, HOIK1, KUCG2, WIBJ2 and WTC; ES cells: H1, H9 and HES3) (around 3 weeks; Extended Data Fig. 3a). Heterogeneity analysis and comparison with a single-cell transcriptomic atlas of the developing mouse brain^6^ revealed transcriptionally distinct clusters organizing along an anterior–posterior axis (Extended Data Fig. 3b). These clusters expressed many transcription factors, secreted ligands and surface receptors associated with patterning centres such as the hypothalamic floor plate (*SIX6*, *HES5*, *SIX3*), roof plate (*FGFR3*, *RSPO3*, *WNT7B*) and hindbrain roof plate (*MSX1*, *BAMBI*, *BNC2*; Extended Data Fig. 3c). Notably, marker expression was consistent between lines; however, cluster proportions varied substantially, consistent with previous reports^25^. We further identified cluster-specific candidate *cis-*regulatory elements (CREs) of patterning-related genes and found that many were similarly accessible across lines (Extended Data Fig. 3d). These data suggest that there is interesting variation between lines in the propensity to self-pattern, and also support a preserved GRN underlying brain region formation.

**Extended Data Fig. 4:**
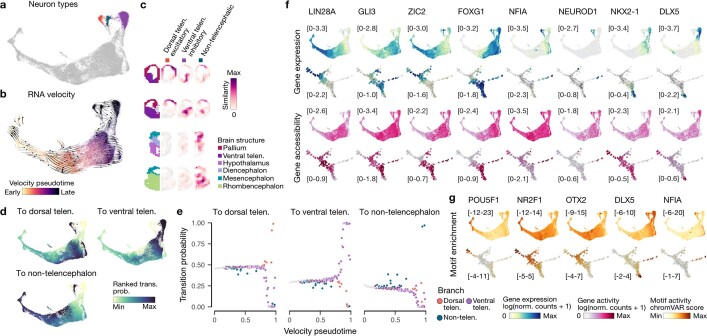
Trajectory reconstruction in the multiomic developmental atlas. a, Time course UMAP embedding coloured by neuron types. b, Time course UMAP embedding coloured by RNA velocity pseudotime. c, VoxHunt plots showing expression similarity of neuron subtypes in brain organoids to voxels in five example sections of the developing mouse brain (embryonic day 13.5), as well as the structural annotation of the sections. d, UMAP embedding coloured by ranked transition probabilities. e, Scatter plot showing mean transition probabilities as computed by CellRank versus velocity pseudotime. Each dot represents one high-resolution cluster. f, UMAP embedding of the integrated time course and graph embedding coloured by gene expression (log(transcript counts per 10k +1)) (top) and gene activity (log(fragment counts per 10k +1)) (bottom) for selected marker genes. g, UMAP and graph representation coloured by transcription factor motif enrichment z-score calculated with chromVAR^59^ for selected motifs. The range of values is indicated for each feature plot.

We next sought to reconstruct the neurogenic differentiation trajectories for each brain region. We used RNA velocity^26,27^ and CellRank^28^ to generate a terminal fate transition probability matrix based on transcriptomes, which we used to construct a differentiation graph of high-resolution metacell clusters and assign branch identities (Fig. 1c and Extended Data Fig. 4a–e). The graph, presented by a force-directed layout, reveals an early bifurcation into anterior telencephalic and posterior non-telencephalic cell states and later branching of telencephalic progenitors into dorsal excitatory and ventral inhibitory neuronal trajectories, respectively (Fig. 1d,e). This telencephalic progenitor state before dorsoventral divergence is marked by the expression of *DCT*, *DIO3* and *SIX6*, and is characterized by transient accessible chromatin regions (Fig. 1f). Transcriptional and regulatory dynamics can be examined along each neurogenic trajectory, revealing regional specificity of gene expression, chromatin accessibility and binding-motif enrichment for stage-specific transcription factors (Fig. 1f and Extended Data Fig. 4f,g). Together, these data provide a multi-omic developmental atlas spanning the course of brain organoid regionalization and neurogenesis.

## Regulatory network inference with Pando

**Fig. 2:**
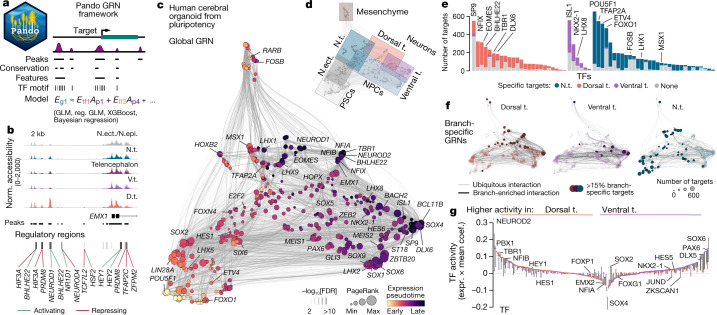
Pando leverages multimodal measurements to infer a multiphasic GRN underlying human brain organoid development. **a**, Schematic of the Pando GRN-inference framework. Candidate regions are identified through intersection of accessible peaks with CREs or conserved elements. Predicted TFs are selected for each candidate region through binding-motif matching. The relationship between TF–binding-site pairs and the expression of target genes is then fitted with a regression model. *E*, expression; *A*, accessibility; g1, target gene 1; tf1,2, transcription factors; p1,4, peaks; GLM, generalized linear model; reg., regularized. **b**, Signal tracks showing normalized accessibility at the transcription start site of *EMX1* in the different branches and inferred regulatory regions for various transcription factors. The line colour represents the sign of the interaction and the box colour (greyscale) represents the false-discovery rate (FDR) of the most significant interaction for this region. **c**, UMAP embedding of the inferred TF network based on co-expression and inferred interaction strength between TFs. Colour and size represent the expression-weighted pseudotime and PageRank centrality of each TF, respectively. **d**, UMAP embedding shaded by module features. **e**, Target specificity for branch-specific TFs. **f**, UMAP embedding of branch-specific TF networks highlighting TFs with branch-specific targets and interactions with branch-specific accessibility. **g**, Groups of TFs with differential activity between the dorsal (red) and ventral (purple) telencephalon branch. TF activity is indicated by a coloured dot for each branch, connected by a line, and was calculated by multiplying the mean regulatory coefficient (coef.) with the average expression (expr.) in the branch. The sign of the activity indicates whether the regulation is mainly activating (+) or repressing (−).

**Extended Data Fig. 5:**
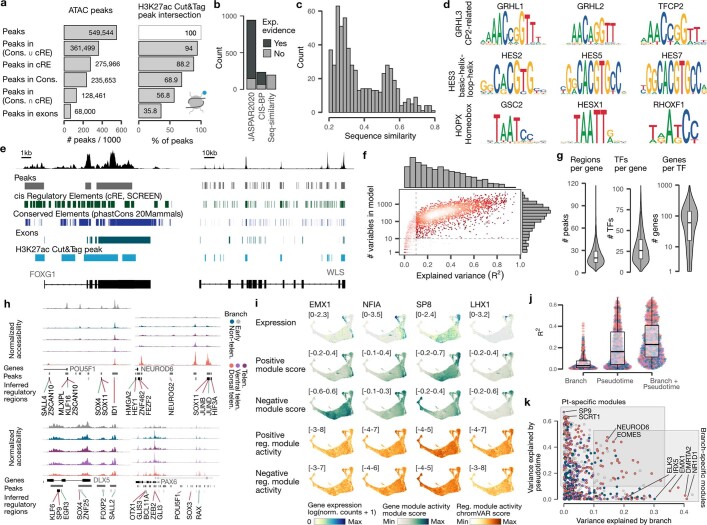
Gene regulatory network features of brain organoid development. a, Numbers of chromatin access peaks and percentage of H3K27ac-marked peaks accessible at day 18–23 (>5% detection) intersecting with non-protein coding conserved regions (Cons.), candidate cis regulatory regions (CRE), or exons (left). b, Representative loci showing chromatin access (top) overlaying peak, CRE, conserved elements, and exon coordinates. c, Barplot showing the number of motifs used in GRN construction from two curated databases (JASPAR, CIS-BP), and motifs assigned through amino acid sequence similarity. d, Examples of 3 TFs with no motif annotation that were assigned motifs based on sequence similarity. e, Loci for two exemplary genes (FOXG1, WLS) showing average chromatin access signal tracks, accessible peaks, CREs, conserved elements, exons and H3K27ac CUT&Tag peaks. f, Scatter plot and histograms show explained variance (x) versus number of variables (y) of models for GRN construction. g, Violin plots show the distribution of peaks (left, n = 2535 target genes) and TFs per gene (middle, n = 2535 target genes), and number of genes per TF (right, n = 720 TFs). h, Representative loci showing average chromatin access signal tracks at different developmental branches overlaying inferred transcription factor binding sites within regulatory regions. i, UMAP representation of time course coloured by gene expression (log(transcript counts per 10k + 1)), gene module activity (module score calculated with Seurat) (rows), and regulatory module enrichment z-score (calculated with chromVAR) for representative TFs (columns). The range of values is indicated for each plot. j, Variation of module activity explained by branch, pseudotime, or branch and pseudotime (n = 720 TF modules). Box plot centre lines represent the median, boxes indicate 25%–75% interquantile range and whiskers 1.5 * interquantile range. k, Branch and pseudotime specific TF modules. Colours represent the branch with highest average module activity. TFs without experimentally validated motif are shown in grey.

To infer the GRN underlying human brain organoid development, we developed an algorithm called Pando (Fig. 2a and Methods), which leverages multimodal single-cell genomic measurements and models gene expression through TF–peak interactions. Pando first identifies candidate regulatory regions that show accessibility across the organoid time course by incorporating information on conservation^29^ and previous CRE annotations^30^ (candidate regions; Extended Data Fig. 5a,b). We performed cleavage under targets and tagmentation (CUT&Tag) analysis of the H3K27ac histone modification marking active promoters and enhancers to assess regulatory region selection performance. We found that 94% of accessible peaks intersecting with H3K27ac were among the candidate regions, indicating a strong enrichment for active regulatory regions (Extended Data Fig. 5a). Next, candidate regions are assigned to genes in their vicinity and TF-binding sites are predicted for each region (Extended Data Fig. 5c–e). Linking regulatory regions to genes on the basis of proximity has limitations; however, it is an effective assumption for many regulatory interactions at the genome scale^31,32^, and we observed a strong correlation between gene expression and a regulatory domain that includes proximal promoter and gene body regions (Extended Data Fig. 1i). Pando then uses a regression model to infer the relationship between the expression of each target gene, TF expression and binding-site accessibility (Fig. 2a and Extended Data Fig. 5f). As a consequence, Pando jointly infers sets of positively or negatively regulated target genes (gene modules) as well as regulatory genomic regions (regulatory modules) for each TF (Fig. 2b and Extended Data Fig. 5g–i). We visualized the GRN using a UMAP embedding, which revealed groups of TFs that are involved in different phases of brain organoid development, broadly representing the pseudotemporal order of cell state transitions (Fig. 2c). A series of TFs tracked transitions from pluripotency (such as POU5F1, LIN28A) to neuroepithelium induction (for example, SOX2 and HES1), with additional module neighbourhoods linked to brain regional NPC specification and neuron differentiation (Fig. 2d and Extended Data Fig. 5j,k). Nodes associated with initializing (pluripotency) and terminal states (regionalized neurons) had a high degree of centrality, reflecting the high number of correlated expressed genes for these states. We found that certain TF modules were pseudotime-dependent independent of brain regional identity (such as SP9, SCRT1), whereas others showed specificity for a given brain region (for example, EMX1, NR1D1, NEUROD6 in the dorsal telencephalon; IRX5 in non-telencephalon) (Extended Data Fig. 5j,k). Globally, this GRN shows that regulatory region accessibility and TF expression track with stages of organoid development and segregate during brain regionalization.

To better understand how chromatin accessibility constrains and specifies GRN activity in different brain organoid regions, we next analysed the differential accessibility of inferred binding sites between regional branches. We pruned regulatory edges with strongly depleted accessibility and could identify TFs with highly branch-specific target sets (Fig. 2e). We further partitioned the global GRN into branch-specific GRNs (Fig. 2f), representing subgraphs of which the activity is shaped by changes in chromatin accessibility between branches. Within these subgraphs, we computed TF activity as the mean coefficient of all active connections multiplied by the mean expression in the branch (Fig 2g). Comparing TF activity in the dorsal and ventral telencephalon branch revealed TFs with high branch specificity (such as NEUROD2, NFIA, SOX6) as well as TFs of which the mode of regulation changed between mainly activating (positive activity) to mainly repressing (negative activity; for example, HEY1, JUND, ZKSCAN1) and vice versa (such as SOX2). Together, these analyses provide a rich resource for future research to understand the gene regulatory programs controlling human brain regionalization and TF-mediated cell programming.

## Single-cell TF perturbations in organoids

**Fig. 3:**
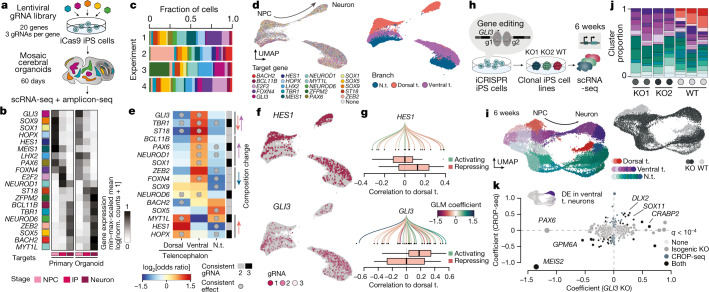
TF perturbations in mosaic organoids reveal critical regulators of neurodevelopmental fate decisions. **a**, Schematic of the single-cell TF perturbation experiment using the CRISPR droplet sequencing (CROP-seq) method. **b**, The minimum–maximum-scaled average expression (log[transcript counts per 10,000 + 1]) of targeted genes in NPCs, IPs and neurons of the primary and organoid cortex. **c**, The proportion of cells with each perturbation for each experiment. **d**, UMAP embedding with cells coloured by detected gRNA (left) and branch assignment (right). **e**, Regional enrichment of gRNAs. The sidebar shows the number of gRNAs that were consistent and the circles represent consistent effects between experiments and statistically significant (FDR < 0.01) effects on composition. The arrows indicate the predominant observed effect. **f**, UMAP embedding coloured by consistent gRNAs for selected genes that had a strong effect on fate regulation. **g**, The Spearman correlation of HES1-target (top, *n* = 18 genes) and GLI3-target (bottom, *n* = 42 genes) genes to transition probabilities into the dorsal branch. The GRN was subsetted to retain connections that are accessible at the branchpoint (>5% detection rate). The centre line represents the median, the box limits show the 25–75% interquartile range and the whiskers indicate 1.5× the interquartile range. **h**, Schematic of the GLI3 loss-of-function experiment using an inducible CRISPR–Cas9 nickase system. **i**, UMAP embedding of scRNA-seq data from 6-week-old WT and *GLI3*-KO brain organoids showing the trajectories from NPCs to neurons coloured by different clusters assigned to regional branches. The inset is coloured by genetic condition. **j**, Stacked bar plots showing the distribution of cluster (colour) assignment per organoid for each condition. **k**, Differential expression (DE) in ventral telencephalic neurons for *GLI3*-KO data and CROP-seq data containing a *GLI3* gRNA. The *x* and *y* axes indicate the coefficients of the linear model. Colours indicate significance (FDR < 10^−4^) in CROP-seq, the KO cell line or both.

**Extended Data Fig. 6:**
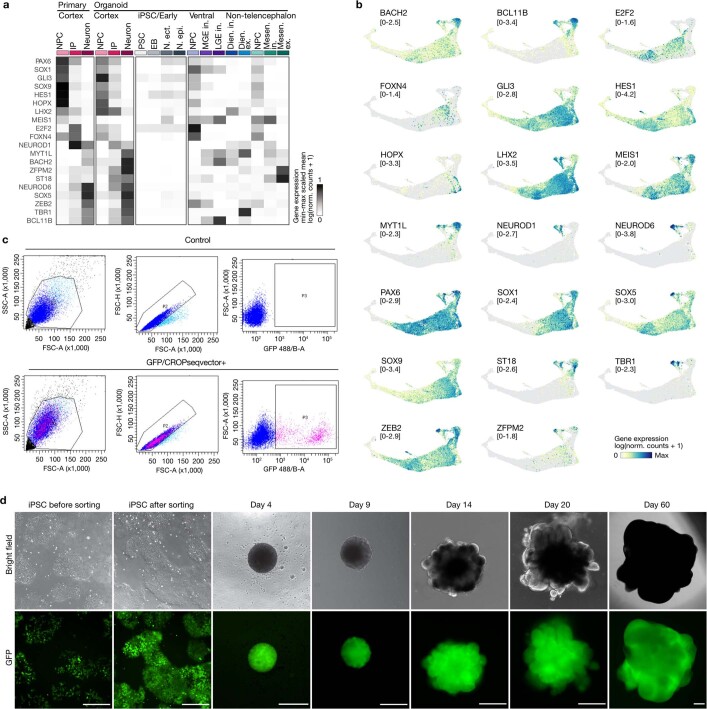
Target selection and experimental details for the single-cell *in organoid* perturbation experiment. a, Min-max scaled mean expression (log(transcript counts per 10k + 1)) of genes targeted in the single-cell genomic perturbation experiment in neuronal progenitors (NP), intermediate progenitors (IP) and neurons in the primary human and organoid developing cortex, as well as in iPS cells, the embryoid body (EB), ventral telencephalic NPCs, inhibitory neurons of the medial ganglionic eminence (MGE in.), lateral ganglionic eminence (LGE in.), non-telencephalic NPCs, diencephalic excitatory neurons (Dien. ex.) and inhibitory neurons (Dien. in.) and mesencephalic excitatory neurons (Mesen. ex.) and inhibitory neurons (Mesen. in.). b, UMAP embedding coloured by the expression of all targeted genes. The range of expression values is indicated for each feature plot. c, Exemplary Fluorescence-activated cell sorting plots of the sorting scheme used to isolate CROP-seq vector positive iPS cells. d, Phase contrast and CROP-seq vector positive (GFP) imaging during brain organoid development. Images are representative for 48 imaged organoids. Scale Bar is 500 µm.

**Extended Data Fig. 7:**
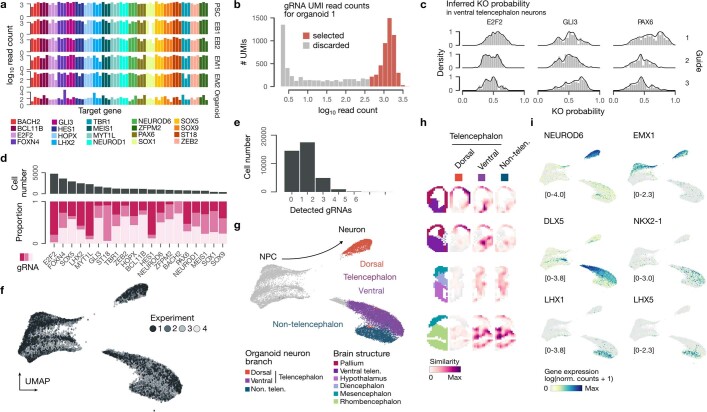
Guide detection and cell type annotation in the single-cell perturbation experiment in organoids. a, Barplot showing number of cells with detected guide RNA (gRNA) for each targeted gene and stacked barplot showing the distribution of the different gRNAs targeting the same gene. b, Histogram showing the distribution of read counts for gRNA UMIs after amplicon sequencing for one organoid. UMIs marked in red were selected for downstream analyses. c, Density histograms showing the distribution of inferred KO probabilities for gRNAs of 3 different target genes. d, Barplot showing cell number and proportion of gRNAs for all target genes. e, Barplot showing the number of guides detected in sequenced cells. f, UMAP embedding with cells coloured based on experiment. g, UMAP embedding coloured by annotated neuron subtypes. h, VoxHunt plots showing expression similarity of neuron subtypes in brain organoids to voxels in five example sections of the developing mouse brain (embryonic day 13.5), as well as the structural annotation of the sections (left). i, UMAP embedding coloured by expression (log(transcript counts per 10k + 1)) of non-telencephalic (top), ventral (middle) and dorsal (bottom) neuron markers. The range of expression values is indicated for each feature plot.

To begin to understand the mechanisms regulating cell fate and state during human brain development, we used a pooled perturbation screen^17^ in mosaic organoids (Fig. 3a). We designed gRNAs and generated a pooled lentiviral library targeting 20 TFs (each targeted by 3 gRNAs) expressed in different stages of both organoid and primary developing human cortex^7^ and with no expression in iPS cells or the neuroectoderm stages (Fig. 3b and Extended Data Fig. 6a,b). We transduced iPS cells containing an inducible *Cas9* cassette with the lentiviral gRNA library, and sorted and expanded vector-positive iPS cells on the basis of fluorescence (Extended Data Fig. 6c). We induced *Cas9* expression in the infected iPS cells expressing different gRNAs, and used the mosaic pool of iPS cells to generate mosaic brain organoids containing a multitude of perturbed genotypes. Fluorescence was maintained throughout organoid development, and bulk amplicon sequencing revealed relatively homogenous detection of the gRNAs (Extended Data Figs. 6d and 7a). At day 60, at which neural progenitors and neurons coexist in the organoid and all targeted TFs have been or are being expressed (Fig. 3b and Extended Data Fig. 6a,b), we dissociated the mosaic organoids and sequenced single-cell transcriptomes and guide cDNA amplicons of three individual organoids as well as a pool of multiple organoids. We recovered 22,449 cells with an assigned gRNA. Each gRNA for all 20 targets was detected at an average of 1 gRNA detected per cell (Fig. 3c and Extended Data Fig. 7b–e). We generated a UMAP embedding, analysed cell type heterogeneity, and annotated NPCs, intermediate progenitors and neurons in the dorsal telencephalon, the ventral telencephalon as well as in non-telencephalic developing brain regions (Fig. 3d and Extended Data Fig. 7f–i).

**Extended Data Fig. 8:**
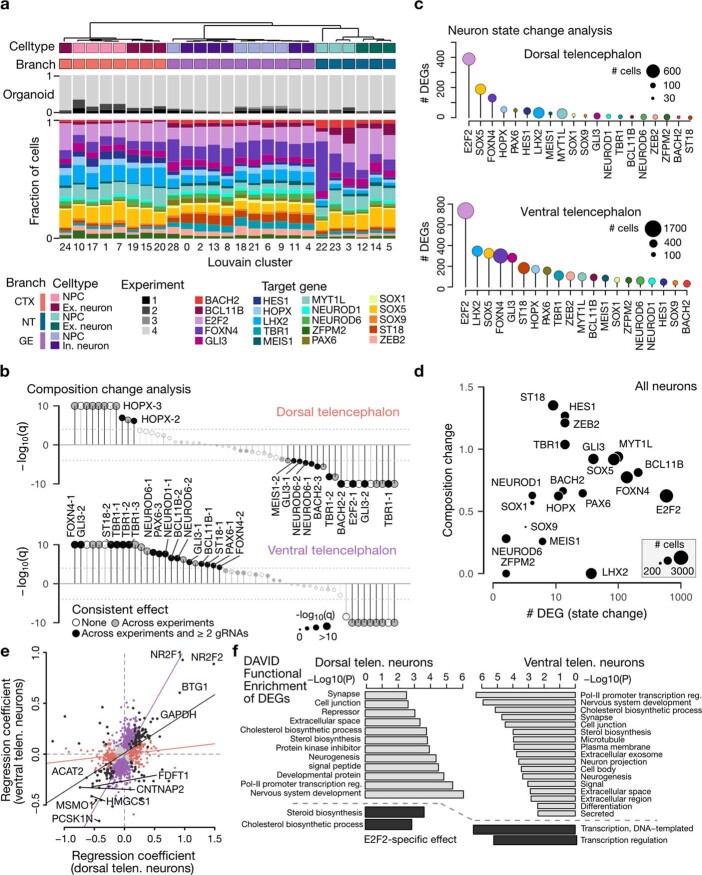
Composition and expression changes after CRISPR-Cas9 perturbations in mosaic brain organoids. a, Hierarchical clustering of Louvain clusters based on the composition of gRNAs targeting different genes. Cell type and branch annotations are shown as side bars. Compositions of organoids and composition of cells with gRNAs targeting different genes are shown below as stacked bar plots. b, Lollipop plot showing the impact of each gRNA on cell type abundance in dorsal and ventral telencephalic neurons. c, Lollipop plots showing number of differentially expressed genes (DEG) for targeted genes in the dorsal and ventral telencephalic neurons. d, Differential gene expression analysis was performed to identify potential effects on cell state. Plot shows the effect of cell composition change and the number of differentially expressed genes (DEGs). P-values were derived using an F-test based ANCOVA. e, Scatter plot shows expression changes between neurons with E2F2 targeting gRNAs and other neurons in dorsal (x-axis) and ventral (y-axis) telencephalic neurons, with each dot representing one gene. Colours of dots represent the neuron types where differential expression is detected. Lines show the correlation of expression changes in the two neuron types, with DE genes in both types and DE genes in only one type shown separately. f, Examples of functional enrichment for E2F2 DEGs in dorsal and ventral neurons with DAVID. Grey bars show enriched terms of all E2F2 DEGs, and dark bars show enriched terms of DEGs with E2F2-specific effects.

We tested the association of gRNA detection on cell type abundance and on differential gene expression within cell types (Extended Data Fig. 8). We first hierarchically clustered Louvain clusters on the basis of gRNA abundance and observed grouping by brain region (Extended Data Fig. 8a). This showed that different brain regions exhibited unique gRNA compositions, suggesting region-specific effects of TF perturbations. We next stratified the detected gRNAs using a log-transformed odds ratio (*P* value based on a Cochran–Mantel–Haenszel test) and assessed the consistency of the effect across organoids and gRNAs (Extended Data Fig. 8b and Supplementary Table 5). On the basis of these metrics, we found that gRNAs targeting eight TFs showed consistent enrichment in the ventral telencephalon branch with corresponding depletion in the other regions, including the cortex (Fig. 3e; for example, *GLI3*, *TBR1*). Another set of perturbations showed the opposing effect, with enrichment of TF targeting gRNAs in the cortex and depletion in either the ventral telencephalon or non-telencephalon (such as *HES1*,* HOPX*). We focused on *HES1* and *GLI3*, two genes that are expressed at the dorsoventral branchpoint and show opposing effects on dorsal telencephalon commitment (Fig. 3e,f). Both genes are known regulators of mouse cortical development^33–35^ and are associated with developmental disorders in humans^14,36^. We used the GRN inferred from the developmental time course to investigate how *GLI3*- and *HES1*-target gene expression is correlated with transition probabilities into dorsal telencephalon (Fig. 3g). We found that genes activated by GLI3 were positively correlated with cortical transition probabilities, whereas *HES1* had a repressive effect on such genes. This suggests an antagonistic involvement of these two genes in shaping the dorsoventral fate decision in the human telencephalon. Notably, we also found that, for several TFs, perturbation led to detectable transcriptomic effects rather than composition changes (Extended Data Fig. 8c–f and Supplementary Tables 6 and 7). In particular, *E2F2*—a crucial cell cycle regulator^37^—altered the transcriptome of both dorsal and ventral telencephalic neurons, suggesting that misregulation of cell cycle exit has a substantial effect on the neuronal transcriptome state. Together, these data provide one of the first implementations of a multiplexed perturbation experiment in organoids to examine the effect of genetic perturbations on human brain cell fate and state development.

## GLI3 directly targets HES regulomes

**Extended Data Fig. 9:**
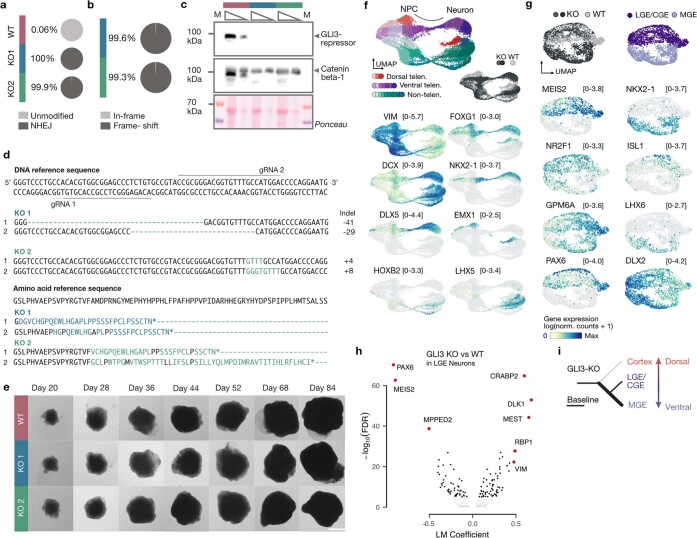
Characterization of GLI3 knock-out organoids. a, Quantification of editing frequency as determined by the percentage and number of reads showing unmodified and modified alleles for the control and both KO cell lines. b, Frequency of frameshift of coding sequence reads as a result of the modifications seen in both KO lines. c, Western blot showing expression of Gli3-repressor (83kDA) in the control cell line. Catenin beta-1 and Ponceau were used as loading control. For western blot source data, see Supplementary Fig. 1 d, Sequences of the coding strand of the different indels of the different KO lines. The reference sequence is corresponding with the control line. The position of the gRNAs with the protospacer adjacent motif (PAM)-sequence is depicted above and underneath the sequence. Reference protein sequence with the protein sequences of each KO line of the altered protein sequences caused by the frame-shift. e, Brightfield images of brain organoid development with control and both KO cell lines. Images are representative for 16 imaged organoids per line. Scale bar is 2 mm. f, UMAP embedding showing trajectories from neural progenitor cells (NPCs) to neurons coloured by different clusters assigned to branches (dorsal, ventral, and non-telencephalon), with inset coloured by genetic condition and feature plots coloured by expression (log(transcript counts per 10k + 1)) of cell type markers. g, UMAP embedding of ventral telencephalic GLI3 KO neurons showing medial ganglionic eminence (MGE) and lateral/caudal ganglionic eminence (LGE/CGE) neuronal populations (top). Feature plots show selected marker gene expression on the UMAP embedding. The range of expression values is indicated for each feature plot. h, Volcano plot showing differential expression analysis in LGE neurons for GLI3 WT versus KO cells. i, Schematic of observed effect of GLI3 loss of function on dorsoventral telencephalic fate decisions.

Mosaic perturbations suggested that GLI3 is involved in dorsoventral neuronal fate specification in the human telencephalon. GLI3 is a well-known mediator of SHH signalling^38^, with GLI3 loss-of-function mutations resulting in the failure of the cortex to form in mice, and the expansion of ventral telencephalic neuronal identities into dorsal locations within the developing brain^39,40^. In humans, mutations in *GLI3* are associated with Greig cephalopolysyndactyly syndrome and Pallister Hall syndrome, in which patients have variable presentations of brain malformations depending on the particular mutations^14^. To confirm that GLI3 is involved in cell-fate establishment in the human context, and to examine the underlying developmental mechanisms, we used CRISPR–Cas9 gene editing to generate two independent *GLI3-*knockout (KO) iPS cell lines and a control wild-type (WT) cell line that went through the editing process (Fig. 3h and Extended Data Fig. 9a–d). We generated KO and WT brain organoids and confirmed that the GLI3 protein is not detected in the KO organoids (Extended Data Fig. 9c, e). We performed scRNA-seq analysis of KO and WT organoids at day 45, a time point of early neurogenesis, and analysed the cellular heterogeneity (Fig. 3i and Extended Data Fig. 9f). Notably, KO cells were depleted in the dorsal telencephalon, with a strong enrichment in the ventral telencephalon (Fig. 3j), and differential gene expression analysis revealed that *GLI3* KO affects ventral telencephalic cell states (Fig. 3k and Supplementary Table 8). Both of these observations were consistent with the mosaic perturbation experiment.

Interestingly, the TF *MEIS2*, a marker of lateral/caudal ganglionic eminence (LGE/CGE) relative to medial ganglionic eminence (MGE), was strongly downregulated in *GLI3*-KO conditions (Fig. 3j). Further analysis of the ventral telencephalic neuron heterogeneity identified distinct LGE/CGE-like and MGE-like neuronal populations with *GLI3-*KO cells strongly enriched in MGE neurons (Extended Data Fig. 9g,h). We observed expression alterations in *GLI3*-KO LGE-like neurons compared with the WT LGE state; genes involved in dorsoventral patterning (*PAX6*, *MEIS2*, *DLK1*) were differentially expressed (Extended Data Fig. 9h). These data confirm that GLI3 is necessary for cortical neuron fate establishment in humans, and its absence affects ventral telencephalon development by promoting MGE neurogenesis and altering LGE neuronal expression, consistent with a role in MGE fate repression^41^ and LGE neuron state regulation (Extended Data Fig. 9h,i).

**Fig. 4:**
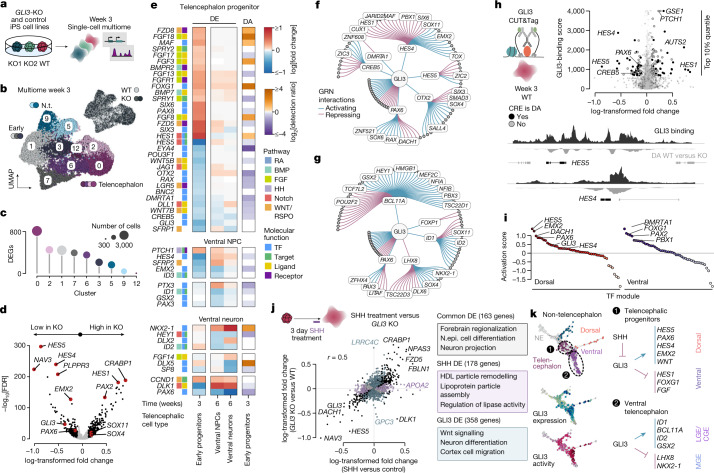
Single-cell multiome view of GLI3 loss of function reveals distinct regulomes and effectors of dorsoventral telencephalon specification. **a**, Schematic of the experiment measuring the transcriptome and chromatin accessibility in the same cell at 3 weeks of brain organoid development. **b**, UMAP embedding coloured by cluster and labelled by projected cell fate. Inset: UMAP coloured by genetic state. **c**, The number of DEGs of control (WT) versus *GLI3-*KO cells in the different clusters. **d**, Differential expression in telencephalic progenitors (clusters 0 and 2) after *GLI3* KO. **e**, DEGs after *GLI3* KO for early telencephalic progenitors (week 3), ventral telencephalic progenitors (week 6) and neurons (week 6), and differential accessibility after *GLI3* KO in early telencephalic progenitors (week 3). Genes are coloured according to the associated signalling pathway (if applicable) and molecular function. **f**,**g**, GRN subgraph for early telencephalic (**f**) and ventral telencephalon (**g**) progenitors, showing first- and second-order GLI3 targets. The circles represent genes for which all TFs are labelled. The edges are coloured on the basis of TF regulatory interaction. **h**, The GLI3-binding score (the sum of CUT&Tag signal intensity for the gene body + 2 kb) in WT organoids versus log-transformed fold change in differential expression in early telencephalic progenitors (week 3). Genes with differentially accessible (DA) CREs are coloured black. Signal tracks of GLI3 binding matched with differential accessibility peaks of HES4 and HES5 in early telencephalic progenitors. **i**, The *z*-scored mean correlation between module gene expression and branch probabilities (branch activation score) for differentially expressed TFs. **j**, The log-transformed fold change of genes after treatment with SHH versus *GLI3* KO. GO terms are shown for common DEGs, SHH-treatment-specific and GLI3-specific DEGs. **k**, Schematic summarizing the results from the GLI3 and SHH perturbations.

**Extended Data Fig. 10:**
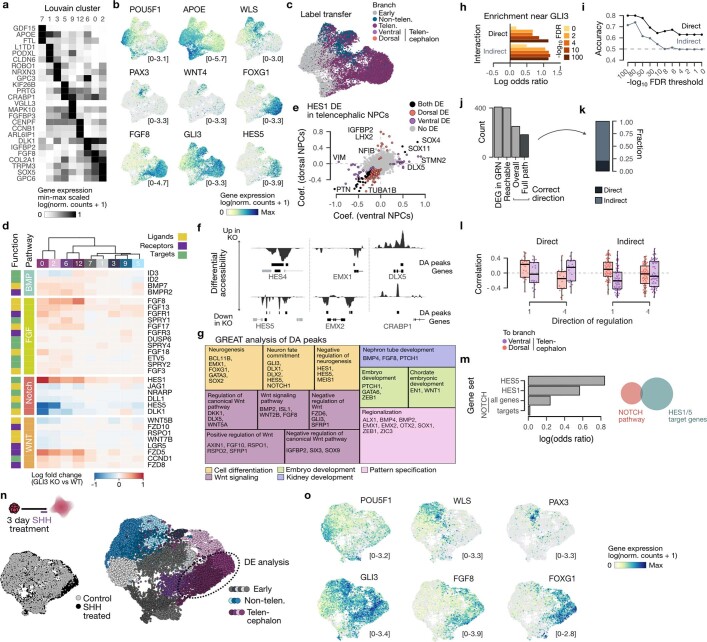
GLI3 KO induced changes in telencephalic progenitors in brain organoids. a, Heatmap showing min-max scaled expression (log(transcript counts per 10k +1)) of marker genes for unbiased Louvain clusters. b, UMAP coloured by the expression of selected marker genes. The range of values is indicated for each plot. c, UMAP embedding coloured by branch labels predicted by label transfer from the organoid time course. d, Heatmap showing DE associated with signalling pathways. e, Scatter plot showing DE in neural progenitor cells (NPCs) upon HES1 perturbation in the mosaic perturbation experiment. f, Signal tracks showing differentially accessible (DA) peaks in cluster 0 and 2. g, GREAT enrichment analysis of DA peaks in cluster 0 and 2, with box area proportional to FDR. Representative genes are shown. h, Enrichment of DE genes in the neighbourhood of GLI3 in the GRN. i, Accuracy of GRN predicted directionality of GLI3 effect at different false discovery rate (FDR) thresholds. j, Barplot showing the number of all DE genes in the GRN (DEG in GRN), all DE genes reachable from GLI3 in the graph (Reachable), DE genes where the GRN was consistent with the DE result (Overall) and DE genes for which all subpaths from GLI3 were consistent with the DE result (Full path). k, Barplot showing the fraction of DE genes directly and indirectly regulated by GLI3. l, Boxplot showing the Spearman correlation of directly (n = 39) and indirectly (n = 126) regulated DE genes with transition probabilities ventral and dorsal branched. The centre line represents the median, boxes indicate 25%–75% interquantile range and whiskers 1.5 * interquantile range. m, Barplot showing the enrichment of gene sets (HES1/5 target genes, NOTCH components) among telencephalic DE genes. n, UMAP embedding showing annotation of multiome SHH experiment. o, UMAP coloured by the expression of selected marker genes.

GLI3 is expressed broadly in progenitors of the telencephalon and of non-telencephalic regions (Extended Data Fig. 4f), suggesting distinct GLI3 regulatory roles during different phases of brain development. We therefore generated single-cell multiome data (10x Genomics) of WT and *GLI3-*KO organoids at a time point (3 weeks) preceding dorsoventral patterning (Fig. 4a and Extended Data Fig. 10a–c). WT and *GLI3*-KO organoids showed comparable cell composition (Fig. 4b); however, strong differential expression and differential accessibility was detected between KO and WT cells in the telencephalic progenitor population (clusters 0 and 2; Fig. 4b,c and Supplementary Table 9). Differentially expressed genes (DEGs) included *HES1* (upregulated) and *HES4* and *HES5* (downregulated) (Fig. 4d and Extended Data Fig. 10d), as well *EMX2* (downregulated). Interestingly, *GLI3*-KO cells showed upregulation of *SOX4* and *SOX11*, two genes detected as downregulated in HES1-perturbed cells in the pooled single-cell perturbation experiment, consistent with the opposing effect of GLI3 and HES1 on dorsal telencephalic fate emergence (Fig. 3e and Extended Data Fig. 10e).

Combining single-cell data of WT and *GLI3-*KO organoids of both time points (3 and 6 weeks) revealed TFs and signalling pathways that are differentially expressed specifically in telencephalic progenitors, ventral telencephalic NPCs and ventral telencephalic neurons (Fig. 4e), hinting towards a distinct regulatory role of GLI3 in these different developmental stages. In telencephalic progenitors, *GLI3* KO leads to the upregulation of FGF-related genes (*FGF8*, *SPRY1*, *FGF13*) and the downregulation of WNT-related genes (*WNT7B*, *WNT5B*, *LGR5*), whereas ventral telencephalic cells showed dysregulation of hedgehog pathway receptor *PTCH1* and several transcription factors including *NKX2-1*, *EMX2*, *GSX2* and *ID1*. *GLI3* KO induced differential accessibility of CREs linked to these genes and pathways (Fig. 4e, Extended Data Fig. 10f,g and Supplementary Tables 10 and 11). Interestingly, many genes were differentially expressed only in the later ventral telencephalic stages, whereas CREs were differential accessibility already in telencephalic progenitors (for example, *NKX2-1*, *ID1*), indicating a potential priming effect.

We investigated the perturbation signatures in the context of our inferred GRN, and observed strong consistency between GLI3 direct and indirect targets and detected DEGs, supporting the predictability of the GRN (Fig. 4f,g and Extended Data Fig. 10h–k). Two GLI3 sub-GRNs describe distinct perturbation effects in telencephalic progenitors and in the ventral telencephalon branch, respectively (Fig. 4f,g). Before dorsoventral fate bifurcation, the sub-GRN suggests that GLI3 directly activates *HES4*, *HES5*, *PAX6*, *OTX2* and *CREB5*, with 76% of the DEGs being indirect targets of GLI3. After specification of the ventral telencephalon, a second sub-GRN suggests that GLI3 directly regulates *PAX6*, *LHX8*, *ID1* and *BCL11A*. GLI3 CUT&Tag analysis in 3-week-old organoids revealed extensive GLI3 binding at genomic regions nearby (*HES4*, *HES5*, *CREB5* and *PAX6*) that also show differential accessibility in *GLI3-*KO cells, confirming that GLI3 binds to these targets directly in telencephalic progenitors (Fig. 4h). Interestingly, even though *HES4/5* can be targets of the Notch pathway, we did not observe enrichment for other NOTCH targets, suggesting the independence of Notch signalling (Extended Data Fig. 10m). We assessed the relevance of GLI3 targets in driving dorsal or ventral telencephalic fate establishment by computing a dorsal and ventral telencephalon branch activation score for each TF module (Fig. 4i, Extended Data Fig. 10l). This analysis suggests that the GLI3 targets *HES5*, *EMX2* and *PAX6* are major drivers of dorsal telencephalic fate, whereas FOXG1 and DMRTA1 activate ventral telencephalic fate.

Finally, we wanted to understand the interplay between GLI3 and SHH—a major inducer of telencephalon ventralization^42,43^. Organoids were treated with SHH for 3 days during the neuroepithelial stage (3 weeks) followed by multiome profiling. Differential expression analysis revealed downregulation of *GLI3* in SHH-treated versus untreated control telencephalic progenitors (Extended Data Fig. 10n,o) and, overall, there was a highly significant correlation with *GLI3-*KO-induced DEGs (Fig. 4j; Pearson’s *r* *=* 0.5). Gene Ontology (GO) analysis showed that shared and GLI3-specific DEGs were enriched in genes related to brain regionalization and differentiation, whereas SHH-specific DEGs were largely lipid-metabolism related. This suggests that SHH promotes ventralization predominantly by preventing GLI3-induced dorsalization^39,44^. Taken together, our data-driven approach provides a multiphasic GLI3 gene regulatory model for human telencephalon development that is consistent with previous studies, while also proposing downstream effectors (Fig. 4k).

## Discussion

The human brain has unique features that distinguish it from other species. Despite the high-resolution descriptions of mouse and human developing brain cell composition from recent cell atlas efforts^4–6^, it has been a major challenge to study the mechanisms that control human brain development owing to the difficulty in obtaining tissue at the earliest stages of brain patterning, and the lack of methods to systematically manipulate gene function. Here we integrated transcriptome, chromatin accessibility and genetic perturbation datasets to provide insights into the mechanisms that underlie human brain regionalization. In a broad sense, we found that the programs identified in mouse and other non-human model systems are well conserved in humans, and the extent that stem-cell-derived brain tissues recapitulate these programs is notable. We focused on GLI3 as a well-studied transcription factor controlling dorsoventral fate specification in the rodent telencephalon. We found clear evidence that this same transcriptional program is well conserved in humans. Importantly, these data provide strong evidence that multiregion human brain organoids can be predictive model systems. Note that unguided neural organoid protocols result in strong variation between stem lines with regard to proportions of regions represented in each organoid or batch.

We established the Pando GRN inference framework, which incorporates features of the regulatory genome that have not previously been used for the global analysis of developmental programs. Pando generalizes regression-based GRN inference for multimodal datasets by combining transcriptome, chromatin accessibility, an expanded TF family motif reference, known CREs and evolutionary conservation into a flexible framework. The R package implements the full GRN inference strategy, including candidate region selection, motif matching, model fitting and discovery of gene and regulatory modules. Furthermore, it offers a wide range of regression models to be used for GRN inference. We have highlighted interesting aspects of the network, such as TF modules involved in the transition from pluripotency through neuroectoderm to a neuroepithelium, as well as the subnetworks associated with regionalized brain states. Such network analysis can guide future experiments designed to program specific neuronal states, and can be used to interpret gene perturbations in human organoids^45^. Note that current limitations include the lack of comprehensive active and repressive histone modification and chromatin conformation status across organoid development, as well as incomplete TF motif databases. We expect these to be an active area of research, and Pando has the flexibility to include such priors into the GRN inference framework.

We validated the critical role of GLI3 in dorsal telencephalic cell-fate specification in humans, and further identified the contribution of GLI3 during specification of MGE and LGE/CGE neurons. The integration of the single-cell multiome data from *GLI3*-KO organoids and the global GRN suggested a model in which GLI3 becomes induced in early telencephalic NPCs through SHH signalling during neuroepithelial regionalization. GLI3 then regulates downstream targets, activating cortical fate acquisition through differential activity of *HES5*, *HES4* and *HES1*, and inhibiting the MGE induction program through regulation of *BCL11A*, *LHX8* and *NKX2-1*. Our data also suggest that GLI3 can regulate HES genes directly, probably through NOTCH-independent mechanisms similar to what has been described recently during mouse limb development^46^. More broadly, our data reveal the extraordinary potential of multimodal single-cell genomic and organoid technologies to understand gene regulatory programs of human brain development.

## Methods

### Experimental methods

#### Stem cell and organoid culture

We used six human iPS cell lines (Hoik1, Wibj2, Kucg2 from the HipSci resource^47^; 409B2 from the RIKEN BRC cell bank; 01F49i-N-B7 (B7) from Institute of Molecular and Clinical Ophthalmology Basel; and WTC from the Allen Institute) and three human ES cell lines (H1-PAX6YFP (H1) and H9 from WiCell and HES-3 NKX2.1GFP/w (HES3) from the Murdoch Children's Research Institute). Stem cell lines were cultured in mTESR1 (Stem Cell Technologies, 05851) with mTeSR1 supplement (Stem Cell Technologies, 05852) and supplemented with penicillin–streptomycin (1:200, Gibco, 15140122) on Matrigel-coated plates (Corning, 354277). Cells were passaged 1–2 times per week after dissociation with TryplE (Gibco, 12605010) or EDTA in DPBS (final concentration 0.5 mM) (Gibco, 12605010). The medium was supplemented with Rho-associated protein kinase (ROCK) inhibitor Y-27632 (final concentration 5 µM, STEMCELL Technologies, 72302) the first day after passage. Cells were tested for mycoplasma infection regularly using PCR validation (Venor GeM Classic, Minerva Biolabs) and found to be negative. A total of 4,500–5,000 cells were plated in ultralow-attachment plates (Corning, CLS7007) to generate brain organoids using a whole-brain organoid differentiation protocol^2^. The use of human ES cells for the generation of brain organoids was approved by the ethics committee of northwest and central Switzerland (2019-01016) and the Swiss federal office of public health.

#### Single-cell RNA-seq, ATAC–seq and multiome experiments for the developmental time course

Brain organoids were generated from four different stem cell lines (H9, 409B2, Wibj2, Hoik1) simultaneously. Brain organoids of the same batch were dissociated at multiple time points of the course of brain organoids development. We collected these single-cell suspensions from an embryoid body time point (day 4), the time points of neuronal induction (days 7, 9 and 11) and after embedding in Matrigel and starting the neuronal differentiation process (days 12, 16, 18, 21, 26, 31 and 61). Organoids of the four different cell lines were pooled on the basis of size and dissociated together, and the cell lines were later demultiplexed on the basis of the single-nucleotide polymorphism information. Multiple organoids of each line were pooled together to obtain a sufficient number of cells. For the early time points, 15 organoids per cell line were pooled, decreasing this number to minimally 3 organoids for the later time points (Supplementary Table 1). For time points just after Matrigel embedding, Matrigel was dissolved in Cell Recovery Solution (Corning, 354253) for 15 min at 4 °C. The organoids were cut in halves and washed three times with HBSS without Ca^2^^+^ and Mg^2^^+^ (STEMCELL Technologies, 37250). Single-cell suspensions were acquired by dissociation of the organoids with a papain-based dissociation (Miltenyi Biotec, 130-092-628). Prewarmed papain solution (2 ml) was added to the organoids and incubated for 15 min at 37 °C. Enzyme mix A was added before the tissue pieces were triturated 5–10 times with 1,000 μl wide-bore and P1000 pipette tips. The tissue pieces were incubated twice for 10 min at 37 °C with trituration steps in between and after with P200 and P1000 pipette tips. Cells were filtered with consecutively with 30 µm and 20 µm preseparation filters and centrifuged. Cells were resuspended and viability and cell count were assessed using a Trypan Blue assay on the automated cell counter Countess (Thermo Fisher Scientific). Cell suspensions were split in two and resuspended in CryoStor CS10 (STEMCELL Technologies, 07952) and cryopreserved at −80 °C. The next day, cryotubes were transferred to liquid nitrogen for storage until the scRNA-seq and scATAC-seq experiments were performed.

The cryopreserved single-cell suspensions of each time point were thawed by warming up the cryo for 1–2 min in a water bath at 37 °C and directly centrifuged in 10 ml prewarmed DMEM with 10% FBS. Cells were washed twice with PBS + 5% BSA and filtered through a 40 µm cell strainer (Flomi). For scATAC–seq, nuclei were isolated according to the protocol provided by 10x genomics (Demonstrated protocol CG000169 Rev D) using the low-input protocol and a lysis time of 3 min. Nuclei were loaded at a concentration that would result in the recovery of 10,000 nuclei. In case of less nuclei recovered, the maximum number of nuclei was targeted. scATAC–seq libraries were generated using the Chromium Single Cell ATAC V1 Library & Gel Bead Kit. Before sequencing, an additional clean-up step was performed to enrich shorter fragments by applying a double-sided (1.2–0.75×) clean-up with AMPureXP beads (Beckman Coulter) and Illumina Free Adapter Blocking Reagent was used to reduce potential index hopping. The libraries were sequenced on the Illumina NovaSeq platform.

For scRNA-seq, cells were put in a concentration after counting and viability checking that enabled targeting 10,000 cells and, in case the cell number was not sufficient, all cells were loaded. scRNA-seq libraries were generated using the Chromium Single Cell 3′ V3 Library & Gel Bead Kit. Single-cell encapsulation and library preparation were performed according to the manufacturer’s protocol.

Single-cell multiome datasets were generated from day 15 brain organoids of the stem cell lines Wibj2, Hoik1, 409B2, B7 and WTC, and day 19 brain organoids of stem cell lines (Kucg2, WTC, B7, and H1 and HES-3 NKX2.1GFP/w) using the Chromium Single Cell Multiome ATAC + Gene Expression kit. Before nucleus isolation, organoids were dissociated with the papain-based dissociation. Nuclei were isolated according to the protocol provided by 10x genomics (demonstrated protocol CG000365, Rev B) in the lysis buffer with final amount of 0.01% Tween-20 and 0.01% Nonidet P40 Substitute and a lysis time of 3 min. Single-cell encapsulation and library preparation were performed according to the manufacturer’s protocol.

Libraries were pooled, FAB treated and sequenced on the Illumina NovaSeq platform. A summary of all single-cell experiments is provided in Supplementary Table 1.

#### Immunohistochemistry

Organoids were washed in PBS before fixing in 4% PFA at 4 °C overnight. The samples were washed three times with PBS and the organoids were then transferred to a 30% sucrose solution for 24–48 h for cryoprotection. Finally, organoids were transferred to plastic cryomolds (Tissue Tek) and embedded in OCT compound 4583 (Tissue Tek) for snap-freezing on dry ice. For immunohistochemical stainings, organoids were sectioned in slices of 10 µm thickness using a cryostat (Thermo Fisher Scientific, Cryostar NX50). Organoid sections were quickly washed in PBS to remove any residual OCT and post-fixed in 4% PFA for 15 min at room temperature. The sections were then incubated in antigen-retrieval solution (HistoVT One, Nacalai Tesque) at 70 °C for 20 min. Excess solution was washed away with PBS and the tissue was incubated in blocking-permeabilizing solution (0.3% Triton X-100, 0.2% Tween-20 and 5% normal donkey serum in PBS) for 1 h at room temperature. Next, the sections were incubated overnight at 4 °C in blocking-permeabilizing solution containing mouse anti-SOX2 (1:200, Sigma-Aldrich, AB5603), rabbit anti-TUJ1 (1:200, BioLegend, 801201) and goat anti-GLI3 (1:200, Novus Biological, AF3690) antibodies. The next day, the sections were rinsed three times in PBS before incubation for 1 h at room temperature with 1:500 secondary antibody (donkey anti-rabbit Alexa 488, ab150073 and donkey anti-mouse Alexa 568, ab175472 and donkey anti-goat Alexa 647, ab150135) in blocking-permeabilizing solution. Finally, the secondary antibody solution was washed off with PBS and the sections were stained with DAPI before covering with ProLong Gold Antifade Mountant medium (Thermo Fisher Scientific). Stained organoid cryosections were imaged using a confocal laser scanning microscope, and six different *z*-plane images (*z*-step = 2–3 µm) were acquired using a ×20 magnification objective. The images were further processed using Fiji.

#### Whole-mount HCR RNA-FISH

Probe sets, amplifiers and buffers were ordered from Molecular Instruments. HCR in situ hybridization was performed according to the manufacturer’s instructions by Molecular Instruments with small changes. In brief, 19-day-old organoids were washed once with PBS and transferred to a tube containing fresh 4% PFA at 4 °C and were fixed overnight at 4 °C. The samples were washed three times with PBST and then dehydrated with a PBST–methanol gradient (25%, 50%, 75% to 100%) and stored at −20 °C in 100% methanol until use. The samples were rehydrated with a similar series of graded methanol–PBST washes for 5 min each on ice and washed an additional time with PBST. The samples were then treated with 10 µg ml^−1^ proteinase K (Invitrogen, 25530-049) for 3 min at room temperature. The samples were washed twice with PBST for 5 min and then post-fixed with 4% PFA for 20 min at room temperature and subsequently washed three times with PBST for 5 min. The organoids were prehybridized in probe hybridization buffer for 30 min at 37 °C. Then, 1 pmol of each probe set was diluted into probe hybridization buffer and the samples were incubated overnight at 37 °C. The samples were washed four times with probe wash buffer at 37 °C and washed twice more with 5× SSCT. The organoids were incubated in amplification buffer for 10 min at room temperature before adding the precooled hairpin mixture diluted in amplification buffer to incubate overnight at room temperature. The excess hairpins were removed with three 5 min washes as well as two longer washes of 30 min. Organoids were stained with DAPI during one of the 30 min washes. The samples were stored at 4 °C and mounted on a µ-Slide chamber (Ibidi, 80807) and covered with 1% agarose. Images were acquired with lambda scanning followed by spectral unmixing on the Zeiss LSM980 system and processed using Fiji.

#### Doxycycline-inducible *Cas9* nuclease and nickase cell line

The human iPS cell line 409B2 was used to create an iCRISPR–Cas9 nickase (Cas9n) and an iCRISPR–Cas9 line as described previously^48^. The doxycycline-inducible *Cas9-*expressing cell line was generated by introducing two transcription activator-like effector nucleases (TALENs) targeting the *AAVS1* locus, which has shown to be effective for sustained transgene expression, and two TALEN constructs with donor plasmids. One of the donor plasmids contained a constitutive reverse tetracycline transactivator (AAVS1-Neo-M2rtTA) and the other one contained a doxycycline-inducible expression cassette (Puro-Cas9). A D10A mutation was introduced by site-directed mutagenesis of the original Puro-Cas9 donor using the Q5 mutagenesis kit (New England Biolabs, E0554S) to generate the *Cas9n*. The cell lines used were tested for the proper expression of pluripotency markers SOX2, OCT4, TRA-1-60 and SSEA, quantitative PCR confirmed the doxycycline-inducible *Cas9n* and digital PCR was used to exclude off-target integration^49^. Both cell lines showed normal karyotypes after generation, but the iCRISPR–Cas9 line acquired a common stem cell abnormality over time. A total of 55% percent of the cells showed a derivative chromosome 2 with a long arm of chromosome 1 (bands q11q44) attached to the long arm of one chromosome 2 (band q37).

#### Vector and lentivirus preparation for the perturbation experiment

The perturbation experiment was performed according to the CROP-seq protocol as described previously^17^ with some small alterations. The experiment was performed in organoids derived from the inducible Cas9 nuclease line, which contains a Puro selection marker. To be able to select for cells that received the CROP-seq vector, Puro was exchanged for eGFP to isolate cells by fluorescence. We selected targeted TFs that had previously been shown in the literature to have correlated expression patterns during human cortex development in organoids and primary tissues, and have been studied in vertebrate models and shown to be involved in regulating forebrain development. The selected TFs had minimal expression in iPS cells and neuroectoderm stages to minimize the chances that organoid development was impaired during the early stages of organoid development. All of the selected TFs were expressed in the organoid dorsal telencephalon, and most were also expressed in at least one other branch. Three gRNA per targeted gene were designed by Applied Biological Materials and synthesized by IDT as 74 base oligonucleotides with 19 and 35 bases of homology to the hU6 promoter and guide RNA backbone, respectively. Oligonucleotides were pooled in equal amounts and were assembled in the vector backbone by Gibson’s isothermal assembly. The plasmid library was sequenced to validate the complexity of the pooled plasmid library. We used 10 ng of plasmid library for generating a sequencing library with a single PCR reaction. Illumina i7 and i5 indices were added by PCR and the library was sequenced on the Illumina MiSeq platform. After validation, lentiviruses were generated by the Viral Core Facility of Charité Universitätsmedizin Berlin.

#### Generation of mosaic organoids for perturbation experiment

The iCRISPR–Cas9 line was cultured on Matrigel in mTesr1 supplemented with penicillin–streptomycin (1:200) and *Cas9* was induced 2 days before lentiviral transduction by adding 2 µg ml^−1^ doxycycline. Then, 24 h later, cells were dissociated into single cells with TrypLE and 300,000 cells of the iCRISPR–Cas9 cells were plated in at least 12 wells of Matrigel-coated 6-well plates in mTesr1 supplemented with penicillin–streptomycin (1:200), Y-27632 (final concentration 5 µM) and 2 µg ml^−1^ doxycycline. Next, 24 h later, cells were transduced with a low multiplicity of infection (MOI) where less than 30% of the cells were GFP^+^ to ensure that the majority GFP^+^ cells received only one lentivirus per cell. The viral particles were added to the culture medium (mTesr1 supplemented with penicillin–streptomycin, Y-27632 and 2 µg ml^−1^ doxycycline). Then, 24 h later, the medium was exchanged for mTesr1 supplemented with penicillin–streptomycin and 2 µg ml^−1^ doxycycline until 70% confluency was reached. Cells were then sorted with fluorescence-activated cell sorting (FACS) for GFP^+^ cells to enrich for CROP-seq-vector-positive cells and plated on Matrigel-coated plates in mTesr1 supplemented with 100 μg ml^−1^ Primocin (InvivoGen, ant-pm-1) and Y-27632 (final concentration 5 µM). When cells reached 70% confluency, whole-brain organoids were generated as mentioned previously.

#### Preparation of single-cell transcriptomes from mosaic perturbed organoids

After 2 months, single organoids and a pool of four organoids were dissociated using a papain-based dissociation kit (Miltenyi Biotec, 130-092-628) as described previously. Cells were sorted using FACS. Cell viability and number was assessed using the Trypan Blue assay and the Countess automated cell counter (Thermo Fisher Scientific). Finally, cells were diluted to an appropriate concentration to obtain approximately 7,000 cells per lane of the 10x microfluidic chip. scRNA-seq libraries were generated using the Chromium Single Cell 3′ V3 Library & Gel Bead Kit. The expression libraries were FAB-treated and sequenced on the Illumina NovaSeq platform.

#### gRNA detection from single-cell cDNA

gRNA were amplified from 60 ng of cDNA remaining from scRNA-seq preparation with three separate PCR reactions similar to reactions described previously^50^. First, cDNA was amplified using PCR broadly targeting the outer part of the U6 promoter. Subsequently, the inner portion of the U6 promoter adjacent to the guide sequence and a TruSeq Illumina i5 adapter. Finally, we added Illumina sequencing i7 adapters. PCRs were monitored using quantitative PCR to avoid overamplification and, after every PCR reaction, the samples were purified using SPRI beads (Beckman Coulter) and libraries were sequenced at 1:10 proportion of the transcriptome library on the Illumina NovaSeq system.

#### gRNA detection from gDNA

Cells from different stages of the organoid protocol were collected (iPS cell, embryoid body, embedded organoids and organoids day 30). QuickExtract (30–60 µl, Epicentre, QE0905T) was added to the cell pellets or organoids and the suspension was incubated at 65 °C for 10 min, 68 °C for 5 min and 98 °C for 5 min to extract the DNA. The same PCR was used to validate the library complexity of the plasmid library^17^. The PCR was performed using the KAPA2G Robust PCR Kit (Peqlab, 07-KK5532-03) using the supplied buffer B and 5 µl isolated DNA. The following program was used: 95 °C for 3 min; 35 cycles of 95 °C for 15 s, 65 °C for 15 s and 72 °C for 15 s; 72 °C for 60 s. Libraries were sequenced using the Illumina MiSeq system (Nano kit) .

#### *GLI3*-KO and control line generation

Two days before lipofection, iPS cell medium was supplemented with 2 µg ml^−1^ doxycycline (Clontech, 631311) to induce *Cas9n* expression. Two guides were designed using the Broad Institute’s CRISPR design tool (http://crispr.mit.edu/). The following guide pair was selected: ACAGAGGGCTCCGCCACGTGTGG, CCGCGGGACGGTGTTTGCCATGG. The Alt-R CRISPR–Cas9 System (IDT) was used for guide delivery with lipofection according to the manufacturer’s protocol. To form the crRNA–tracrRNA complex in a 3 µM final concentration for each guide complex, 1.5 µl of each guide crRNA was combined with 3 µl tracrRNA and 44 µl nuclease-free water. For the reverse transfection, 1.5 µl of the crRNA–tracr complex mix and 0.75 µl RNAiMAX (Invitrogen, 13778075) were diluted in 47.75 µl OPTI-MEM (Gibco, 1985-062) for each replicate and incubated for 20 min at room temperature in a well of 96-well plate coated with Matrigel (Corning, 35248). During incubation, around 70% confluent cells were detached with TryplE (Gibco, 12605010), centrifuged and resuspended in 1 ml mTeSR with Y-27632 (final concentration 10 µM, STEMCELL Technologies, 72302). After complex incubation, cells were diluted 30 or 60 times in 100 µl mTeSR with Y-27632 (STEMCELL Technologies, 72302) and 2 µg ml^−1^ doxycycline (Clontech, 631311) and the cell suspension was added to a well containing the transfection complexes. After 24 h, the medium was replaced with mTeSR1 medium and cells were allowed to recover for 72 h. Wells at 70% confluence were used for further processing after 72 h. Cells were passaged as single cells in a Matrigel-coated (Corning, 35248) six-well plate in mTeSR medium with 1:200 penicillin–streptomycin (Gibco, 15140122) and Y-27632 (STEMCELL Technologies, 72302). Low amounts of cells were plated per well to avoid the fusion of colonies. The medium was changed daily and Y-27632 was added for the first 72 h to prevent apoptosis of the single cells. When colonies were apparent, single colonies were picked by scraping with a 10 μl pipette tip. Two-thirds of the cell suspension was plated in a single well of a Matrigel-coated 96-well plate in mTeSR1 supplemented with 1:200 penicillin–streptomycin and Y-27632. The other portion of the cell suspension was pelleted and used for validation of frameshift mutations by sequencing. Validated clones were expanded, cryopreserved and karyotyped. The three selected lines, one WT and two KO lines, showed a normal karyotype.

#### Validation of KO lines by sequencing

The cell pellets of picked colonies were resuspended in 10 µl QuickExtract (Epicentre, QE0905T) and the suspension was incubated at 65 °C for 10 min, 68 °C for 5 min and 98 °C for 5 min to extract the DNA. A PCR reaction was performed with primers containing Illumina sequencing adapters for the targeted locus of the *GLI3* gene. Amplification was performed using the KAPA2G Robust PCR Kit (Peqlab, 07-KK5532-03) using the supplied buffer B and 2 µl of extracted DNA. The following program was used: 95 °C for 3 min; 35 cycles of 95 °C for 15 s, 65 °C for 15 s and 72 °C for 15 s; and 72 °C for 60 s. Unique P5 and P7 Illumina indices were added to 0.5 µl of the previous PCR product with a second PCR program (98 °C for 30 s; 25 cycles of 98 °C for 10 s, 58 °C for 10 s and 72 °C for 20 s); and 72 °C for 5 min), using the Phusion HF MasterMix (Thermo Fisher Scientific, F-531L). The double-indexed libraries were pooled and purified with SPRI beads. Purified libraries were sequenced on the MiSeq (Illumina) system resulting in paired-end sequences of 2 × 150 bp. LeeHom^51^ was used to trim the adapters after base calling using Bustard (Illumina).

#### Western blotting

*GLI3* WT and KO organoids of day 15 were collected into Laemmli buffer, homogenized with a pestle (Fisherbrand, 12-141-368) and sonicated for 15 cycles using the Bioruptor Plus. Subsequently, two high and low amounts of protein extractions and ladder (Thermo Fisher Scientific, 26620) were run on an 8% SDS–PAGE (Bio-Rad System) and transferred to a PVDF membrane using Wet-Blot. After blocking for 20 min with 4% milk powder in PBS + 0.1% Tween-20, the primary antibody (1:1,000, stock 0.5 µg µl^−1^, R&D systems, AF3690) was incubated overnight at 4 °C. After washing three times for 7 min at room temperature in PBS + 0.1% Tween-20 on a shaker, the secondary goat IgG HRP-conjugated antibody (1:7,000, R&D systems HAF017) diluted in 4% milk in PBS + 0.1% Tween-20 was incubated for 2 h. The enhanced chemiluminescence signal was recorded using the ChemiDoc system. The loading control β-catenin (primary antibody: stock 1:10,000, Cell Signaling technologies, L54E2; secondary antibody: stock 0.8 µg µl^−1^ 1:7,000, Jackson ImmunoResearch, 115-035-003) was probed on the same membrane and loading was also controlled by Ponceau staining. Raw images are provided in Supplementary Fig. 1.

#### Generation of single-cell transcriptome and multiome of *GLI3-*KO and WT organoids

Organoids of *GLI3* WT and KO iPS cells were generated simultaneously and dissociated with a papain-based dissociation kit (Miltenyi Biotec, 130-092-628) as described above. scRNA-seq was performed on day 45 of organoid development for both KO lines and the WT line for two independent organoid batches. After dissociation, cell viability was checked, cells were counted and 7,000 cells were targeted per lane of the 10x microfluidic chip. Libraries were generated with the Chromium Single Cell 3′ V2 Library & Gel Bead Kit and sequenced on the Illumina HiSeq platform.

Combined scRNA-seq and scATAC-seq were generated using the Chromium Single Cell Multiome ATAC + Gene Expression kit. In the case of SHH treatment, *GLI3* WT organoids were treated with or without 200 ng ml^−1^ SHH (R&D systems, 1845-SH-025/CF) every day for three days before the experiment on day 19. *GLI3*-KO and WT organoids were dissociated with the papain-based dissociation kit on day 19. Nuclei were isolated according to the protocol provided by 10x genomics (demonstrated protocol CG000365, Rev B) with a lysis time of 3 min. The gene expression and accessibility libraries were FAB-treated and sequenced on the Illumina NovaSeq platform.

#### Bulk CUT&Tag for GLI3 and H3K27ac

Single-cell suspensions of 18- or 23-day-old brain organoids were prepared using the Miltenyi Neural Tissue Dissociation Kit (P) (Miltenyi Biotec, 130-092-628) according to the manufacturer’s guidelines. Cells were counted and directly transferred into CUT&Tag Wash buffer supplemented 0.01% digitonin (20 mM HEPES pH 7.5; 150 mM NaCl; 0.5 mM spermidine; 1× Roche protease inhibitor cocktail). Per experiment, 1.5 million cells were used and incubated with 1.5 µg anti-GLI3 antibodies (R&D systems, AF3690) or 1 µg anti-H3K27ac antibodies (Diagenode, C15410196). All of the following steps were performed as described previously^52^. The protein A-Tn5 was purified in house as described previously^52^. The final libraries were sequenced on the NovaSeq platform with paired-end 2 × 50 bp read length.

### Data analysis methods

#### Preprocessing of scRNA-seq data from the organoid time course

We used Cell Ranger (v.3.0.2) with the default parameters to obtain transcript count matrices by aligning the sequencing reads to the human genome and transcriptome (hg38, provided by 10x Genomics, v.3.0.0). Count matrices were further preprocessed using the Seurat R package (v.3.2)^19^. First, cells were filtered on the basis of unique molecular identifier (UMI) counts, the number of detected genes and the fraction of mitochondrial genes. The threshold of mitochondrial gene fraction was held constant across datasets (<0.2). As sequencing depth varied between time points, the threshold of UMI count and number of detected genes was set individually for each sample as follows: days 4 and 7: #UMI: >10,000, <80,000; #features: >3,000, <8,000; day 11: #UMI: >10,000, <60,000; #features: >3,000, <8,000; day 12: #UMI: >2,500, <40,000; #features: >1,000, <6,000; day 16: #UMI: >10,000, <60,000; #features: >3,000, <8,000; days 18 and 21: #UMI: >2,500, <60,000; #features: >1,500, <8,000; day 26: #UMI: >2,500, <60,000; #features: >2,000, <8,000; day 31: #UMI: >2,500, <50,000; #features: >2,400, <7,500; day 61: #UMI: >1,000, <60,000; #features: >1,000, <8,000.

Transcript counts were normalized to the total number of counts for that cell, multiplied by a scaling factor of 10,000 and subsequently natural-log transformed (NormalizeData()).

#### Preprocessing of scATAC–seq data from the organoid time course

We used Cell Ranger ATAC (v.1.1.0) with the default parameters to obtain fragment files by aligning the sequencing reads to the human genome and transcriptome (hg38, provided by 10x Genomics, v.1.1.0). Peaks were called from the fragment file using MACS2 (v.2.2.6). Both the fragment files and the peak count matrices were further preprocessed using Seurat (v.3.2)^19^ and Signac (v.1.1)^53^. First, peaks were filtered by width (<10,000 bp, >20 bp) to retain only high-quality peaks. Furthermore, the following quality control metrics were computed using Signac: a transcription start site (TSS) enrichment score (TSSEnrichment()), nucleosome signal (NucleosomeSignal()), the percentage of reads in peaks and the ratio of reads in genomic blacklist regions. Subsequently, cells were filtered based on the following metrics: percentage of reads in peaks > 30%; number of peak region fragments > 5,000; blacklist ratio < 0.003; nucleosome signal < 5; number of TSS fragments > 5,000; TSS enrichment score > 2.

We then created a unified set of peaks from the union of peaks from all of the samples by merging overlapping and adjacent peaks. The unified set of peaks was requantified for each sample using the fragment file (FeatureMatrix()). Peak counts were normalized by term frequency–inverse document frequency (tf-idf) normalization using the Signac functions RunTFIDF().

#### Demultiplexing of different lines based on single-nucleotide variant information

Cells pooled from different stem cell lines were demultiplexed using demuxlet^54^. Genotyping information was called using bcftools based on (sc)RNA-seq (B7, H1 and HES3) or DNA-seq data (H9 and 409B2)^25,55^ or downloaded from the HipSci (WIBJ2, HOIK1) or Allen Institute (WTC) website. All files were merged using bcftools and sites with the same genotypes in all of the samples were filtered out. Demuxlet was run with default settings. Cells with ambiguous or doublet assignments were removed from the data. For all other cells, the best singlet assignment was considered.

#### Integration of transcriptome and chromatin accessibility data

To create a shared feature space between the two modalities, gene activities were calculated from chromatin accessibility data using the Signac function GeneActivity() with the default parameters and subsequently log-normalized with a scaling factor of 10,000. For each time point and line separately, we performed CCA on gene activities and gene expression data using the Seurat function RunCCA() based on 2,000 features, which were selected using the Seurat function SelectIntegrationFeatures(). In CCA space, we performed minimum-cost maximum-flow (MCMF) bipartite matching between the modalities as described previously^20^ (https://github.com/ratschlab/scim). The function get_cost_knn_graph() was used with knn_k = 10, null_cost_percentile = 99, capacity_method=’uniform’ and otherwise the default parameters. On the basis of the bipartite matches, matched cells were summarized to metacells containing measurements from both modalities. If multiple cells from one modality were included in a metacell, the arithmetic mean between cells was calculated.

#### Removal of cells with glycolysis signature

An additional quality-control step was applied at the level of metacells to remove cells with transcriptomic signatures of glycolysis upregulation. This was based on primary cell type predictions using public human fetal brain scRNA-seq data (Nowakowski dataset)^4^. We fit a multinomial logistic regression model with lasso regularization penalty (alpha = 1), using gene expression ranks of the Nowakowski dataset as the training set, to predict the cell type identity of metacells in the organoid developmental time course. Metacells that were predicted to be of ‘glycolysis’ identity were excluded from the dataset. To fit the logistic regression model and automatically determine the regularization parameter lambda through cross-validation, we used the function cv.glmnet() from the glmnet R package.

#### Integration of different lines and time points

Integration of lines and time points was performed using the log-normalized gene expression data of metacells. To select a set of features suitable for integration of all lines and time points, we selected the union of the 100 most variable genes for each time point separately (local) as well as across the full dataset (global). Analogously, we selected the union of locally and globally variable transcription factors (Supplementary Table 2). We used the union of the selected genes and TFs and further excluded cell-cycle-related genes^56^ from the set. Next, we computed cell cycle scores using the Seurat function CellCycleScoring(). Subsequently the data were *z*-scaled, cell cycle scores were regressed out (ScaleData()) and Principal Component Analysis (PCA) was performed using the Seurat function RunPCA(). We used the first 10 principal components (PCs) to integrate the different time points in the dataset using the CSS method^21^. To remove any remaining cell cycle signal for any downstream tasks, we again regressed out the cell cycle scores from the integrated CSS matrix. To obtain a two-dimensional representation of the data, we performed UMAP^57^ using RunUMAP() with spread = 0.5, min.dist = 0.2 and otherwise the default parameters.

#### Calculation of motif enrichment scores

Position weight matrices of human TF-binding motifs were obtained from the CORE collection of JASPAR2020^58^. Motif positions in accessible chromatin regions were determined using the R package motifmatchr (v.1.14) (10.18129/B9.bioc.motifmatchr) through the Signac function FindMotifs(). Enrichment scores of motifs in accessible regions were calculated for each metacell using chromVAR^59^ through the Signac function RunChromVAR().

#### RNA velocity calculation

To obtain count matrices for the spliced and unspliced transcriptome, we used kallisto (v.0.46.0)^60^ by running the command line tool loompy fromfastq from the Python package loompy (v.3.0.6) (https://linnarssonlab.org/loompy/). Spliced and unspliced transcriptomes were summarized to the metacell level as described above. RNA velocity was subsequently calculated using scVelo (v.0.2.2)^27^ and further analysed using scanpy (v.1.7.0)^61^. First, 2,000 highly variable features were selected using the function scanpy.pp.highly_variable_genes(). Cell cycle genes^56^ were excluded from this feature set and the dataset was subsetted to the resulting gene set. Subsequently, moments were computed in CSS space using the function scvelo.pp.moments() with n_neighbors = 30. RNA velocity was calculated using the function scvelo.tl.velocity() with mode=‘stochastic’ and a velocity graph was constructed using scvelo.tl.velocity_graph() with the default parameters. To order cells in the developmental trajectory, a root cell was chosen randomly from cells of the first time point (day 4) and velocity pseudotime was computed with scvelo.tl.velocity_pseudotime(). The obtained velocity pseudotime was further rank-transformed and divided by the total number of metacells in the dataset.

#### Annotation of organoid developmental stages

To annotate different organoid developmental stages, we first divided the dataset into 20 bins on the basis of quantiles of velocity pseudotime. For each bin, we computed the average gene expression and peak accessibility across metacells and computed the pairwise Pearson correlation between log-normal gene expression values of each bin. From the correlation coefficient *r*, we defined a distance metric as 1 − *r* and used it to perform hierarchical clustering using the ward.D2 method as implemented in the stats R package (hclust()). On the basis of the resulting clusters, bins were manually annotated as PS cells, neuroectoderm, neuroepithelium, NPCs or neurons.

#### Identification of stage-specific chromatin access

To find sets of peaks with stage-specific accessibility, we computed for each stage the percentage of metacells in which each peak was detected. We then computed a specificity score by dividing the detection percentage for each stage by the detection percentage of all other metacells. We filtered peaks with an in-stage detection percentage of >15% and a stage specificity of >1.5. From these peaks, we selected the top 5,000 peaks with the highest specificity score. Using these specific peak sets for each stage, we used GREAT^31^ with the GRCh38 genome assembly and otherwise the default parameters to obtain functional enrichment results. We reported GO biological process enrichments with FDR < 0.01 and that were supported by >30 foreground regions.

#### Inference of regional cell-fate trajectories

To resolve the regional cell fate branches we relied on CellRank (v.1.3.0)^28^ to compute transition probabilities into terminal cell states and PAGA^62^ to obtain a graph abstraction of the transcriptomic manifold. First, terminal neuronal states were annotated manually using VoxHunt (v.1.0.0)^8^ based on the top 20 structure markers. To resolve the developmental trajectories leading up to the emergence of neurons with distinct regional identities, transition probabilities to each of the terminal states were computed for each cell using CellRank. A transition matrix was constructed by combining a velocity kernel (VelocityKernel()) and a connectivity kernel (ConnectivityKernel()) with weights of 0.5 each. Absorption probabilities for each of the predefined terminal states were computed using the GPCCA estimator. From these probabilities, we computed a transition score by ranking the absorption probabilities and normalizing by dividing by the total number of metacells. We then constructed a graph abstraction of the dataset by high-resolution clustering using the Louvain algorithm^63^ with a resolution of 20. We used PAGA to compute the connectivites between clusters (scvelo.tl.paga()) and summarized transition scores for each of the clusters. To find branch points at which the transition probabilities into different fates diverge, we then constructed a nearest-neighbour graph between the high-resolution clusters based on their transition scores (*k* = 30). We further pruned the graph to retain only edges between nodes with a connectivity score of >0.2 and edges going forward in pseudotime, that is, from a node with a lower velocity pseudotime to a node with a higher velocity pseudotime. The resulting graph is directed with respect to pseudotemporal progression and represents a coarse-grained abstraction of the fate trajectory, connecting groups of cells with both similar transition probabilities to the different lineages and high connectivities on the transcriptomic manifold. To assign fate identities to each branch in the graph, we first selected the nodes with the highest transition probability and pseudotime for each of the terminal states as tips. We then performed 10,000 random walks with 200 steps from each tip along edges backwards in pseudotime using the igraph R package (v.1.2.6) (https://igraph.org/). Next, we computed for each node the visitation frequency from each of the terminal states. We then assigned branch identities to each node on the basis of the visitation frequencies as follows: if a node’s visitation frequency from one tip was more than 100× higher than from the next highest tip, it was unambiguously assigned the identity of this tip. If the visitation frequencies from multiple tips were within 100× of each other, then the node was assigned the identity of all of such tips. Nodes that were assigned both the dorsal telencephalic and ventral telencephalic identity were relabelled as ‘telencephalon’. Nodes that were assigned all three identities were labelled as ‘early’ to indicate that their fate was not yet committed. Nodes that could not be reached through this procedure were assigned the identity of the node with the highest connectivity score. The final labelled graph was visualized using the Fruchterman–Reingold layout algorithm as implemented in the igraph R package.

#### Analysis and integration of multiome data in the neuroepithelial stage

Initial transcript count and peak accessibility matrices were obtained with Cell Ranger Arc (v.1.0.0) and further preprocessed using the Seurat (v.3.2)^19^ and Signac (v.1.1)^64^ R packages. Transcript counts were log-normalized and peak counts were tf-idf-nomalized. On the basis of the RNA modality, the data were integrated with previously described data from the neuroepithelial stage using Seurat CCA integration with the default parameters. PCA was performed on integrated, log-normalized and *z*-scaled transcript counts and Louvain clustering was performed using the Seurat function FindClusters() with a resolution of 0.8.

#### Identification of CREs from multiome data

CREs for genes were discovered by linking peaks to genes by co-accessibility and co-expression between ATAC and RNA modalities, respectively. This was achieved using the Seurat function LinkPeaks() with the default parameters.

#### GRN inference with Pando

We developed Pando to infer GRNs while taking advantage of multimodal single-cell measurements, where both the RNA and the ATAC components are either measured for each cell or integrated to obtain metacells or clusters with both modalities. The core GRN inference algorithm in Pando can be summarized in four main steps:

1. Selecting candidate regulatory genomic regions.
2. Scanning regions for transcription factor binding motifs.
3. Selecting region–TF pairs for each target gene.
4. Constructing a regression model with region–TF pairs as independent variables and the expression of the target gene as the response variable.

The coefficients (or importances) of this model can now be seen as a measure of interaction between the region–TF pair and the downstream gene, resulting in a regulatory graph. In the following sections, we will describe these steps in more detail.

#### Selection of candidate regions for GRN inference

To narrow the set of genomic regions that are taken into account for each target gene when constructing the model, we can take advantage of prior knowledge about the potential importance of these regions. Genomic sequence conservation is one such criterion that indicates the relevance of a stretch of DNA, as it has been maintained by natural selection. Thus, we first intersected the peak regions in the ATAC–seq data with the set of PhastCons conserved elements^29^ from an alignment of 30 mammals (obtained from https://genome.ucsc.edu/). As exonic regions tend to be conserved regardless of their regulatory relevance, we further excluded exonic regions from this set. Furthermore, we considered candidate cCREs derived from the ENCODE project^30^. For this, we obtained the set of all human cCREs from https://screen.encodeproject.org/ (GRCh38) and intersected it with peak regions. The union of the resulting conserved and cCRE regions was carried forward as the set of candidate regions for GRN inference.

#### Construction of an extended motif database for GRN inference

Because TFs need to be matched with potential binding sites, the availability of a binding motif is required for a TF to be included in the GRN. We therefore aimed to gather motif information for all TFs relevant in our dataset. First, we selected the union of the 4,000 most variable genes in each individual time point (Supplementary Table 2). All TFs in this set were considered to be relevant. We then obtained binding motifs from JASPAR (2020 release)^58^ taking into account the CORE and the UNVALIDATED collection. For TFs for which no binding motif was available in JASPAR, we further considered the CIS-BP database^65^. Where possible, motifs with direct experimental evidence were prioritized over inferred motifs and motifs that were inferred based on other JASPAR motifs were prioritized over the rest. For all relevant TFs that were also not covered by CIS-BP, motifs were inferred on the basis of protein sequence similarity to other TFs from the same family. Family information and protein sequences for all TFs were obtained from AnimalTFDB^66^ and pairwise multiple-sequence alignments were performed using the Needleman–Wunsch algorithm^67^ as implemented in needle from the EMBOSS software suite (v.6.5.7)^68^. For each query TF, we considered all TFs from the same family with a global sequence similarity of at least 20% and selected the motifs from the three most similar TFs. TF motifs from all sources were combined into one database and motif positions in accessible chromatin regions were determined using the R package motifmatchr (v.1.14) (10.18129/B9.bioc.motifmatchr) through the Signac function FindMotifs().

#### Coarse-graining expression and chromatin accessibility data

Before inferring the GRN, we coarse-grained the data to denoise and remove sparsity. First, we summarized the expression and chromatin accessibility of close cells using the pseudocell algorithm outlined in ref. ^25^. In brief, we randomly selected 30% of all cells in the dataset as the seed cells and constructed a territory for each seed with the ten nearest neighbours based on Euclidean distances using the top 20 PCs. If one cell was assigned to multiple territories, one was randomly chosen. For all cells contained in a territory, gene expression data were summarized using the arithmetic mean. For chromatin accessibility data, an accessibility probability for each territory was computed by averaging binarized read counts. We further performed latent semantic indexing (LSI) on the peak counts of each territory using the Signac functions RunTFIDF() followed by RunSVD(). On the basis of the top 20 LSI components, we further performed high-resolution clustering using the Louvain algorithm with a resolution of 100 and accessibility probabilities were further summarized to a cluster level by computing the arithmetic mean so that each cell in the cluster was represented by the same vector.

#### Linear model-based GRN inference

Pando used a regression-based approach to infer the regulatory interactions between TF–binding site pairs and the corresponding gene. Although the package implements a variety of regression models, here we used a linear model to perform network inference. Genomic coordinates for all genes were obtained via the R package EnsDb.Hsapiens.v86 (10.18129/B9.bioc.EnsDb.Hsapiens.v86). For each gene, we considered a regulatory region encompassing the gene body and 100 kb upstream of the TSS. We then define a linear model on the log-normalized expression *Y* of the gene *i* based on all TF–binding-site interactions in this region:

$${Y}_{i}\,={\sum }_{j}{{\boldsymbol{\beta }}}_{j}\,{e}_{j}\,{a}_{j}+{\varepsilon },$$

where the log-normalized expression of transcription factor *j* is the accessibility probability of the peak that overlaps its binding region, *β*_*j*_ is the fitted coefficient for this interaction and *ε* is the intercept. The fitted coefficients can then be interpreted as the regulatory effect of TF–binding-site pairs on the downstream genes. To fit the linear model, we use the function glm() from the stats R package using Gaussian noise and an identity link function.

#### Peak and gene module construction

To prune the network and retain only significant interactions, the fitted coefficients were tested for statistical significance using analysis of variance (ANOVA). We corrected for multiple testing using the Benjamini–Hochberg method to obtain an FDR-adjusted *P* value, to which a significance threshold of 0.05 was applied. The remaining connections were further summarized to extract sets of negatively (coefficient < 0) and positively (coefficient > 0) regulated target genes and regulatory regions for each transcription factor.

#### Pando implementation details

Pando was implemented as an R package and is available at GitHub (https://github.com/quadbiolab/Pando). Pando was designed for easy use and integrates smoothly with widely used single-cell analysis tools in R, namely Seurat and Signac. Its core functionality is implemented in four main functions:

initiate_grn() selects candidate regions from the dataset and initiates the object for GRN inference. The user can flexibly define custom sets of candidate regions to be taken into account by Pando.

find_motifs() scans candidate regions for transcription factor motifs. The motif database constructed in this work is included in the Pando package, but can also be manually supplied.

infer_grn() selects regulatory regions for each target gene and performs the model fitting. We implemented support for all generalized linear models provided by the stats R package, regularized linear models provided by the glmnet R package^69^, Bayesian regression models implemented through the brms R package^70^, gradient boosting regression through the xgboost R package^70,71^, as well as bagging and Bayesian ridge models through scikit-learn^72^. Where possible and necessary, we also implemented the appropriate statistical tests to obtain *P* values for the coefficients. For bagging ridge models, coefficients can be tested across estimators using a *t*-test or Wilcoxon rank-sum test. For the Bayesian ridge model, we obtain for each coefficient the mean and s.d. and subsequently calculate a *P* value based on the normal distribution. For Bayesian regression models obtained from brms, we calculated *P* values using the bayestestR R package^73^.

find_modules() constructs gene and regulatory modules for each transcription factor.

The implementation is flexible and enables the user to apply the Pando framework to a wide range of use-cases.

#### Visualization of the GRN

We sought to visualize the inferred transcription factor network based on both co-expression and regulatory relationships between transcription factors. First, we computed the Pearson correlation between log-normalized expression of all transcription factors in the network across all metacells in the atlas. From the correlation value *r* and estimated model coefficient *β* between all transcription factors *i* and *j*, we then computed a combined score *s* as

$${S}_{ij}={r}_{ij}\times \sqrt{| {\beta }_{ij}| }+1$$

resulting in a TF-by-TF matrix. We performed PCA on this matrix and used top 20 PCs as an input for UMAP as implemented in the uwot R package (https://github.com/jlmelville/uwot) with the default parameters.

#### Region-specific GRN

The region-specific GRN was generated by incorporating region-specific accessibility profiles and region-specific TF expression into the Pando-inferred GRN. To get the region-specific accessibility, we first performed outlier analysis on the high-resolution clusters of pseudocells described above when summarizing the peak accessibility probabilities for Pando. In brief, for each predicted TF-binding region, a trimmed *z*-transformation was applied to the accessibility probabilities across clusters, with the average number of ATAC reads per pseudocell in the cluster as the covariate and regressed out. Here, instead of the arithmetic mean and s.d. across all clusters, only clusters with probabilities between the 5th and 95th percentiles were used to calculate the mean and s.d. for the transformation. The resulting *z*-scores were converted to *P* values based on the standard Gaussian distribution, which represents the statistical significance of outlier clusters with significantly lower accessibility (BH-corrected FDR < 0.01, close outliers). Alternatively, the statistical significance of outlier clusters with significantly higher accessibility was represented as 1 − *P* (BH-corrected FDR < 0.01, open outliers). Next, a step-wise procedure was used to define clusters with this region accessible. In brief, if no close outlier was found, the open outliers were considered as clusters with the region accessible. When close outliers were detected, all of the clusters except for the close outliers were considered to have the region accessible. When no outlier was detected, the region was considered to be accessible in all clusters. Next, this region accessibility was propagated from the clusters to the bimodal metacells. Given the regional identity of the metacells, a *χ*^2^ enrichment test was used to identify regions that significantly deplete metacells with the region accessible (two-sided *χ*^2^ test, BH-corrected FDR < 0.01, odds ratio < 0.5, inaccessible regions). For each region, the Pando-inferred TF–target regulation mediated by the inaccessible regions in one region was excluded from the region-specific GRN. Finally, the region-specific GRN was further trimmed by excluding any TF with a detection rate of less than 5% in metacells of the region.

#### Calculation of module activity and analysis of module branch specificity

On the basis of the GRN inferred by Pando, the activity of a transcription factor can be represented by the expression of the set of genes that it regulates (gene modules) or by the accessibility of its set of regulatory regions (regulatory modules). To calculate the activity of gene modules, we used the Seurat function AddModuleScore() with all genes included in GRN inference as the background (pool). For regulatory modules, we used the R package chromVAR (v.1.14)^59^ to obtain a set of background peaks (getBackgroundPeaks()). We then computed deviations in accessibility from the background for each regulatory module (computeDeviations()). Next, we assessed how the activity of positively regulated gene modules varied during neurogenesis over pseudotime and between branches. For this analysis, we excluded all cells from the PSC and neuroectoderm stage. We fit three gaussian linear models for each gene *i* module with module activity (*Y*) as the response variable and branch assignment and/or velocity pseudotime as the independent variables: (1) *Y*_*i*_ ~ branch; (2) *Y*_i_ ~ pseudotime; and (3) *Y*_i_ ~ branch+pseudotime.

We used the *R*^2^ value of these models as the fraction of variance explained by branch (1), pseudotime (2), or branch and pseudotime (3). We further tested for differential module activity between the branches for each branch point separately using a Wilcoxon rank-sum test as implemented in the R package presto^74^. For the comparison of the dorsal and ventral telencephalon, we considered only cells in the top 30% pseudotime quantile (NPC and neuron stages). To visualize dorsal and ventral telencephalon-specific transcription factor networks, we first selected positively regulated gene modules of transcription factors with branch-specific expression (described above). For each branch, we then selected the top 15 modules of which the module activity was significantly upregulated (FDR < 0.05) based on the mean difference of module activity between the branches.

#### Preprocessing, integration and annotation of CROP-seq single-cell RNA-seq data

As with the organoid time course, count matrices were obtained using Cell Ranger (v.3.0.2) and further preprocessed using the Seurat R package (v.3.2)^19^. First, cells were filtered on the basis of UMI counts (>500, <30,000), the number of detected genes (>500, <6,000) and the fraction of mitochondrial genes (<0.1). Transcript counts were normalized to the total number of counts for that cell, multiplied by a scaling factor of 10,000 and subsequently natural-log transformed (NormalizeData()). The different samples were integrated using RSS^25^ based on the 2,000 most variable features (FindVariableFeatures()). In RSS space, we performed Louvain clustering with a resolution of 3. Regional identities as well as NPC/neuron identities were assigned to Louvain clusters using a combination of VoxHunt similarity maps and canonical marker genes. Cells annotated as off-target cell types such as mesenchyme and choroid plexus were removed from all downstream analyses.

#### Assignment of gRNA labels to cells

To assign gRNA labels to cells, reads obtained from amplicon sequencing were first aligned to the human genome and transcriptome (hg38, provided by 10x Genomics), which was extended with artificial chromosomes representing the CROP-seq-Guide-GFP construct^17^, using Cell Ranger. We observed that read counts of gRNA UMIs followed a bimodal distribution, with the lower peak probably representing sequencing or amplification artefacts. To extract the higher peak, we first fit a Gaussian mixture model with two components on natural log-transformed read counts using the function GaussianMixture() from the scikit-learn Python package^72^. We then used a probability cut-off of 0.5 to extract the mixture component with higher average read counts. From these gRNA UMIs, we constructed a cell *x* guide count matrix, which was further binarized to obtain the final cell-to-gRNA assignments.

#### Inference of perturbation probability

To account for a potential mixture of unperturbed and perturbed cells in the population, we inferred probabilities of a gRNA having a phenotypic effect on the cell using the strategy proposed previously^15^. Here a Bayesian approach is used to obtain the probability of a cell being perturbed given the observed transcriptome. To this end, a regression model is fit with the gene expression matrix as the response *Y* and the native gRNA assignments, cell and sample covariates as independent variables (*X*):

$$Y=X{\boldsymbol{\beta }}.$$

After fitting the model, the model fit is re-evaluated for each cell with the gRNA assignment set to 0 (*X*_0_). The difference of the squared errors of the two fits can then be transformed into a probability with:

$$P\left({X}_{j}\,=1\right)={\rm{logistic}}\left(\frac{{\sum }_{i}{[{Y}_{ij}-{X}_{0}{\beta }_{i}]}^{2}-{[{Y}_{ij}-{\hat{Y}}_{ij}]}^{2}}{2{\sigma }^{2}}\right)$$

where $${\rm{logistic}}(x)=\frac{1}{1+{{\rm{e}}}^{-x}}.$$

As in the original publication, we used linear regression model with elastic net regularization (alpha = 0.5) using Gaussian noise and an identity link function to fit the model based on 500 most-variable features. The regularization parameter lambda was automatically determined through cross-validation as implemented in the function cv.glmnet() from the glmnet R package. Models were fit for each gene *i* on log-normalized transcript counts *Y* with binary assignments *X* for each gRNA *j* as well as cell type, sample and number of detected genes as covariates:

$${Y}_{i}\propto {n}_{{\rm{f}}{\rm{e}}{\rm{a}}{\rm{t}}{\rm{u}}{\rm{r}}{\rm{e}}{\rm{s}}}+{\rm{s}}{\rm{a}}{\rm{m}}{\rm{p}}{\rm{l}}{\rm{e}}+{\rm{c}}{\rm{e}}{\rm{l}}{\rm{l}}\,{\rm{t}}{\rm{y}}{\rm{p}}{\rm{e}}+{\sum }_{j}{X}_{j}$$

After computing the above-described perturbation probabilities for each cell and gRNA, they were further summarized to a target gene level by taking the maximum probability among the three gRNAs targeting the same gene.

#### Determination of transcriptomic perturbation effects in the CROP-seq screen

To determine how gene KOs affect the transcriptomic state of neuronal populations arising in brain organoids, we used a linear model-based approach^15^. For each neuronal type, we inferred perturbation probabilities *p* for each target gene *j* as mentioned above and fit a linear model on log-normal transcript counts (*Y*) for each gene i as follows:

$${Y}_{i}\sim {n}_{{\rm{f}}{\rm{e}}{\rm{a}}{\rm{t}}{\rm{u}}{\rm{r}}{\rm{e}}{\rm{s}}}+{\rm{s}}{\rm{a}}{\rm{m}}{\rm{p}}{\rm{l}}{\rm{e}}+{\sum }_{j}{X}_{j}.$$

To determine KO effects in neural NPCs, we also used cell cycle phase as a covariate. For this, we inferred the cell cycle phase with the Seurat function CellCycleScoring() and then constructed the linear model as follows:

$${Y}_{i}\propto {n}_{{\rm{f}}{\rm{e}}{\rm{a}}{\rm{t}}{\rm{u}}{\rm{r}}{\rm{e}}{\rm{s}}}+{\rm{s}}{\rm{a}}{\rm{m}}{\rm{p}}{\rm{l}}{\rm{e}}+{\rm{c}}{\rm{c}}\,{\rm{p}}{\rm{h}}{\rm{a}}{\rm{s}}{\rm{e}}+{\sum }_{j}{X}_{j}.$$

To determine the KO effects across all neurons, we inferred global perturbation probabilities on the full dataset and then fit a linear model across neuronal populations on log-normal transcript counts for each gene *y* as follows:

$${Y}_{i}\propto {n}_{{\rm{f}}{\rm{e}}{\rm{a}}{\rm{t}}{\rm{u}}{\rm{r}}{\rm{e}}{\rm{s}}}+{\rm{s}}{\rm{a}}{\rm{m}}{\rm{p}}{\rm{l}}{\rm{e}}+{\rm{n}}{\rm{e}}{\rm{u}}{\rm{r}}{\rm{o}}{\rm{n}}\,{\rm{t}}{\rm{y}}{\rm{p}}{\rm{e}}+{\sum }_{j}{X}_{j}.$$

The coefficients for each target gene were tested using ANOVA and multiple-testing correction was performed using the Benjamini–Hochberg method to obtain an FDR-adjusted *P* value. Genes for which the coefficient of a target gene were significant (FDR < 10^−4^) were treated as differentially expressed genes for this target gene.

#### Determination of composition changes in the CROP-seq screen

To assess the degree to which the KO of a target gene changes the regional composition of the organoid, we first tested the enrichment of each gRNA in each regional branch. To control for confounding effects through differential gRNA abundance in different organoids, we used a Cochran–Mantel–Haenzel (CMH) test stratified by organoid. Moreover, we performed a Fisher’s exact tests to test for enrichment for each organoid individually. Multiple-testing correction was performed using the Benjamini–Hochberg method. To account for other potential within-sample confounders such as clonal heritage, we first required for each gRNA that the enrichment was significant (FDR < 0.05) in more than one individual organoid and that the direction of each significant enrichment was consistent across organoids. All gRNAs for which this was not the case were removed. In a second step, we further required for remaining gRNAs that the same significant effect (FDR < 0.01) was observed for at least one other gRNA targeting the same gene. For the remaining gRNAs, we summarized the assignments for each target gene *i* and calculated the log odds ratio of the enrichment in each regional branch *j* with

$${{\rm{LOR}}}_{ij}=\log \frac{{N}_{g=i{\rm{;}}b=j}/{N}_{g=i{\rm{;}}b\ne j}}{{N}_{g\ne i{\rm{;}}g=j}/{N}_{g\ne i{\rm{;}}b\ne j}}$$

where *N* is a matrix of cell counts for each target gene in each branch. For each target gene, the maximum log odds ratio across the three branches was treated as a measure of composition change.

#### Preprocessing and integration of single-cell RNA-seq data from the *GLI3*-KO experiment

Transcript count matrices were obtained using Cell Ranger (v.3.0.2) and further preprocessed using the Seurat R package (v.3.2)^19^. First, cells were filtered on the basis of UMI counts (>200, <60,000), the number of detected genes (>200, <6,000) and the fraction of mitochondrial genes (<0.1). Transcript counts were normalized to the total number of counts for that cell, multiplied by a scaling factor of 10,000 and subsequently natural-log transformed (NormalizeData()). From all protein coding, non-mitochondrial and non-ribosomal genes, we selected the 200 most variable based on the vst method (FindVariableFeatures()). PCA was performed based on the *z*-scaled expression of these features. Different samples were integrated using CSS^21^ based on the top 20 PCs with the default parameters. To visualize the dataset in two dimensions, we used UMAP on the CSS coordinates with spread = 0.5, min.dist = 0.2.

#### CRISPResso analysis and protein sequence prediction

To find clones with a frame-shift mutation, CRISPResso was used to analyse the sequencing data^75^. This tool aligned the amplicons to the wild-type gene sequence to call in-frame and frameshift indels. Analyses were performed using the following parameters: -w20, -min_indentiy_score70 and -ignore_substitutions. Substitutions were ignored, only sequences with a minimum of 70% similarity were used and only indels present in a window of 20 bp from each of the gRNAs were called. Cell lines were considered to be KOs when >98% of the reads were considered to be a non-homologous end-joining event, the indels caused a frameshift, not more than two different indels were seen and were present in a 50:50 distribution. The predicted protein sequence was obtained using the Biopython Python package^76^.

#### Preprocessing and integration of multiome data from the *GLI3*-KO experiment

Initial transcript count and peak accessibility matrices were obtained using Cell Ranger Arc (v.1.0.0) and further preprocessed using the Seurat (v.3.2)^19^ and Signac (v.1.1)^64^ R packages. Peaks were called from the fragment file using MACS2 (v.2.2.6) and combined in a common peak set before merging. Cells were filtered based on transcript (UMI) counts (>1,000, <25,000), mitochondrial transcript percentage (<30%), peak fragment counts (>5,000, <700,000) and TSS enrichment score (>1). Transcript counts were normalized to the total number of counts for that cell, multiplied by a scaling factor of 10,000 and subsequently natural-log transformed (NormalizeData()). PCA was performed using the Seurat function RunPCA(). Different samples were integrated based on the top 20 PCs with Harmony^77^ using the function RunHarmony() from the R package SeuratWrappers (v.0.3.0) (https://github.com/satijalab/seurat-wrappers) with max.iter.harmony = 50 and otherwise the default parameters.

#### Annotation of cells from the *GLI3*-KO and SHH experiment

To annotate the cell states from both the scRNA and the multiome experiments, we used the annotations of the annotated multi-omic atlas of organoid development that was previously generated. We transferred the regional branch labels using the method implemented in Seurat using the functions FindTransferAnchors() and TransferData(). We then performed Louvain clustering with a resolution 1 for the scRNA data and 0.8 for the multiome data. Clusters were manually assigned to branch identities based on the transferred labels as well as marker gene expression. In the case of the multiome data, we identified populations of mesenchymal and non-neural ectoderm cells, which were excluded from the downstream analysis.

#### Differential expression analysis for the *GLI3*-KO and SHH experiment

To assess the transcriptomic effects of the *GLI3* KO in ventral telencephalon neurons, we performed differential expression analysis using a linear-model-based approach analogous to the approach used in the CROP-seq screen. We fit a linear model on log-normal transcript counts *Y* for each gene *i* with the KO label and number of detected features as independent variables:

$${Y}_{i}\propto {n}_{{\rm{f}}{\rm{e}}{\rm{a}}{\rm{t}}{\rm{u}}{\rm{r}}{\rm{e}}{\rm{s}}}+{\rm{K}}{\rm{O}}\,{\rm{l}}{\rm{a}}{\rm{b}}{\rm{e}}{\rm{l}}.$$

The coefficient of the KO label was tested using ANOVA. To perform differential expression of KO versus control in the multiome data and treated versus control for the SHH experiment, we performed a Wilcoxon rank-sum test using the presto R package (v.1.0.0)^74^. Multiple-testing correction was applied to all results using the Benjamini–Hochberg method to obtain FDR-adjusted *P* values.

#### Differential accessibility analysis for the *GLI3*-KO experiment

To find peaks with differential accessibility between *GLI3* KO and control, we fit a generalized linear model with binomial noise and logit link for each peak *i* on binarized peak counts *Y* with the total number of fragments per cell and the KO label as the independent variables:

$${Y}_{i}\propto {n}_{{\rm{f}}{\rm{r}}{\rm{a}}{\rm{g}}{\rm{m}}{\rm{e}}{\rm{n}}{\rm{t}}{\rm{s}}}+{\rm{K}}{\rm{O}}\,{\rm{l}}{\rm{a}}{\rm{b}}{\rm{e}}{\rm{l}}.$$

We also fit a null model, where the KO label was omitted:

$${Y}_{i}\propto {n}_{{\rm{f}}{\rm{r}}{\rm{a}}{\rm{g}}{\rm{m}}{\rm{e}}{\rm{n}}{\rm{t}}{\rm{s}}}.$$

We then used a likelihood ratio test to compare the goodness of fit of the two models using the lmtest R package (v.0.9) (https://cran.r-project.org/web/packages/lmtest/index.html). Multiple-testing correction was performed using the Benjamini–Hochberg method.

#### Comparison of perturbation effects with GRN

Before using the GRN to interpret the DE results, we first sought to assess the degree to which the transcriptomic effects of the *GLI3* KO are consistent with the inferred GRN. We tested the enrichment of DE genes in the first (direct) and second order (indirect) neighbourhood of *GLI3* in the GRN graph using a Fisher’s exact test. Furthermore, we computed the shortest path from *GLI3* to every DE gene in the GRN graph. To test how accurately the GRN can be used to predict the directionality of the DE, we computed the combined direction of each path as the product of the signs of all individual edges. We then determined the overall predicted effect of GLI3 on each DE gene by computing the mode of the directions of all shortest paths leading to that gene. We defined accuracy as the fraction of genes for which the DE direction was the inverse of the predicted overall effect. Next, we further filtered the paths so that all paths were composed only of DE genes and the direction of each path and subpath was consistent with the DE direction. To visualize this subgraph, we further pruned the graph by retaining only the path with the lowest average log_10_-transformed *P* value for each DE gene.

#### Functional annotation of differentially accessible genomic regions

To better functionally assess the epigenomic effects of the *GLI3* KO, we performed functional enrichment analysis using GREAT^31^. We performed differential accessibility analysis in clusters 0 and 2 (early telencephalon) and applied an FDR threshold of 10^−4^. From all differentially expressed peaks, we selected the top 5,000 peaks with the lowest (most negative) linear model coefficient (depleted in the KO). We further selected all peaks that were accessible in at least 1% of cells in these clusters as the set of background peaks. Using these two peak sets, we used GREAT with the GRCh38 genome assembly and otherwise the default parameters to obtain functional enrichment results. We reported GO biological process enrichments with FDR < 0.01 and that were supported by >100 foreground regions.

#### Analysis of GLI3 CUT&Tag data

To assess GLI3 binding with CUT&Tag data, we first obtained bigwig files with intensity scores across genomic coordinates. From these scores, we computed a per-gene binding score by summing the intensities over the gene body plus an extended promoter region of 2 kb.

### Statistics and reproducibility

Representative images of organoids in culture are shown from batches with 16–96 organoids per cell line (Extended Data Figs. 1a, 6d and 9c). Immunohistochemistry analysis of SOX2, TUJ1 and GLI3 (Extended Data Fig. 1k) was performed on four different cell lines on 2–3 organoids per cell line from one batch. HCR RNA-FISH (Extended Data Fig. 2g) for BMP7 and WLS was performed on day 18 organoids from four different cell lines with 2–3 organoids per experiment and HCR RNA-FISH for FGF8 and WNT8B was performed on day 18 organoids cell line Wibj2 for 3 organoids.

### Reporting summary

Further information on research design is available in the Nature Research Reporting Summary linked to this article.

## Online content

Any methods, additional references, Nature Research reporting summaries, source data, extended data, supplementary information, acknowledgements, peer review information; details of author contributions and competing interests; and statements of data and code availability are available at 10.1038/s41586-022-05279-8.

## Supplementary information

Supplementary Figure Raw western blot data. The red boxes indicate the cropped areas shown in Extended Data Fig. 9.

Reporting Summary

Supplementary Table 1 Overview of single-cell genomic experiments.

Supplementary Table 2 Feature sets used for integration and GRN inference.

Supplementary Table 3 Stage-specific features.

Supplementary Table 4 GO enrichments for stage-specific genomic regions.

Supplementary Table 5 Enrichment effects in the CROP-seq screen. *P* values were derived using a Cochran–Mantel–Haenzel test and FDR correction.

Supplementary Table 6 Transcriptomic KO effects in the CROP-seq screen. *P* values were derived using ANOVA and FDR correction.

Supplementary Table 7 Functional enrichments for *E2F2-*KO effects. *P* values were derived from DAVID, which uses a a modified one-sided Fisher’s exact test.

Supplementary Table 8 Differential expression in ventral telencephalon neurons after *GLI3* KO. *P* values were derived using ANOVA and FDR correction.

Supplementary Table 9 Differential expression in early telencephalic progenitors after *GLI3* KO. *P* values were derived using ANOVA and FDR correction.

Supplementary Table 10 Differential accessibility in early telencephalic progenitors after *GLI3* KO. *P* values were derived using a likelihood-ratio test and FDR correction.

Supplementary Table 11 GO enrichments for differentially accessible genomic regions in *GLI3* KO. *P* values were derived using a hypergeometric test and FDR correction as well as Bonferroni correction.

## Data Availability

Raw sequencing data are available at ArrayExpress. The accessions for the individual experiments are E-MTAB-12001 for developmental time course scRNA-seq data, E-MTAB-11998 for developmental time course scATAC-seq data, E-MTAB-12004 for multiome data of the neuroepithelial stage, E-MTAB-11999 for scRNA-seq data of the CROP-seq experiment, E-MTAB-12005 for amplicon sequencing of the CROP-seq experiment, E-MTAB-11997 for scRNA-seq data of *GLI3*-KO organoids, E-MTAB-12002 for multiome data of *GLI3-*KO organoids, E-MTAB-12003 for multiome data of SHH-treated organoids and E-MTAB-12006 for CUT&Tag data. Processed data and the VCF files for demultiplexing are available at Zenodo (10.5281/zenodo.5242913).

## Code availability

The Pando R package is available on GitHub (https://github.com/quadbiolab/Pando). Other custom code used in the analyses is deposited on GitHub (https://github.com/quadbiolab/organoid_regulomes).
