# Supplementary material for: Inferring and perturbing cell fate regulomes in human brain organoids
Source: Nature. 2022 Oct 5;621(7978):365–72. doi: 10.1038/s41586-022-05279-8 (PMC10499607; doi:10.1038/s41586-022-05279-8)
Supplement: Supplementary file 1 — Raw western blot data. The red boxes indicate the cropped areas shown in Extended Data Fig. 9. [file 41586_2022_5279_MOESM1_ESM.pdf]

nature portfolio

https://doi.org/10.1038/s41586-022-05279-8

Supplementary information

Inferring and perturbing cell fate regulomes in human brain organoids

In the format provided by the authors and unedited

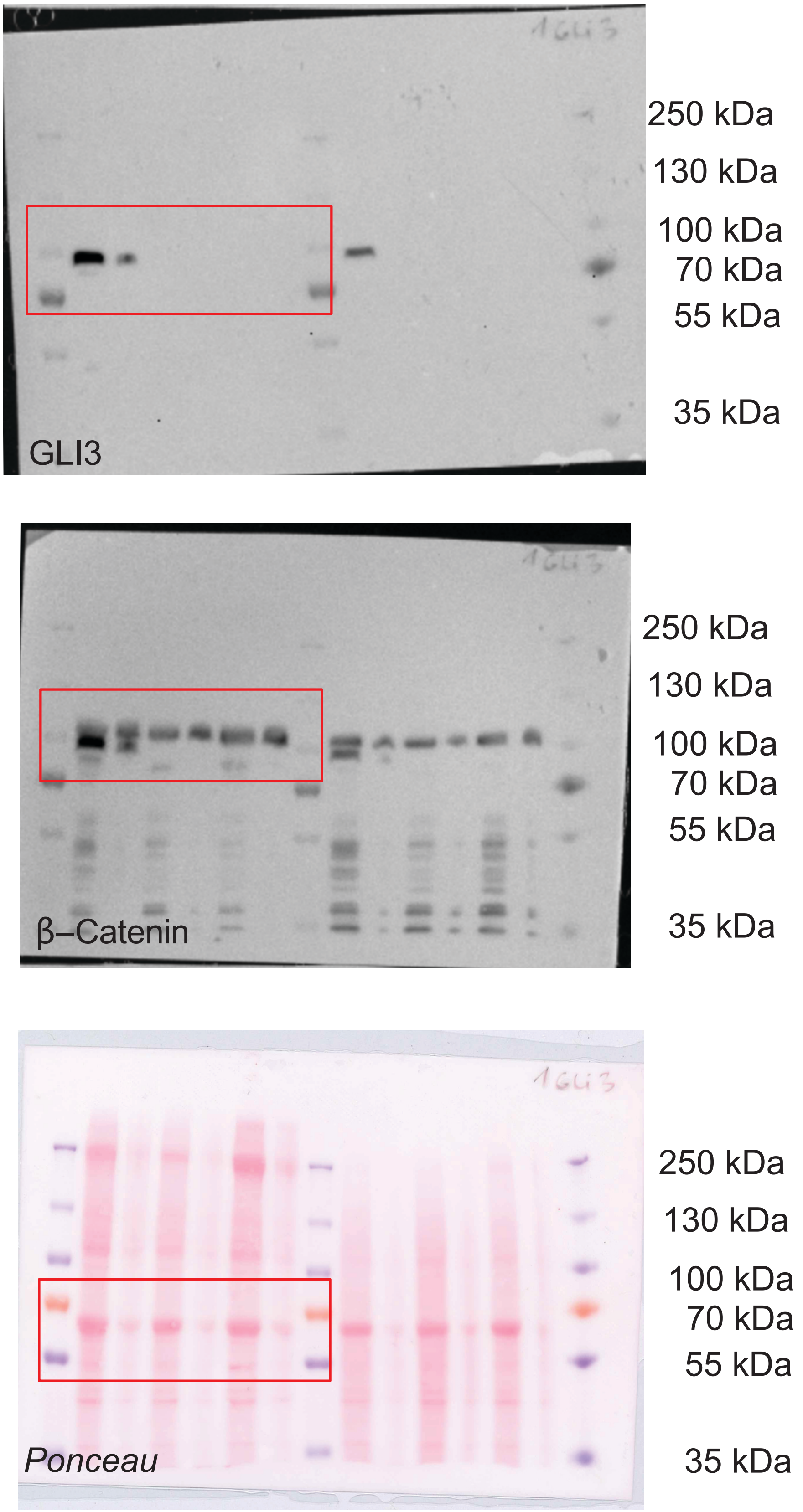
GLI3
250 kDa
130 kDa
100 kDa
70 kDa
55 kDa
35 kDa
β–Catenin
250 kDa
130 kDa
100 kDa
70 kDa
55 kDa
35 kDa
Ponceau
250 kDa
130 kDa
100 kDa
70 kDa
55 kDa
35 kDa
