# Supplementary material for: Inferring and perturbing cell fate regulomes in human brain organoids
Source: Nature. 2022 Oct 5;621(7978):365–72. doi: 10.1038/s41586-022-05279-8 (PMC10499607; doi:10.1038/s41586-022-05279-8)
Supplement: Supplementary file 2 — Reporting Summary [file 41586_2022_5279_MOESM2_ESM.pdf]

nature portfolio

Corresponding author(s): Zhisong He
J. Gray Camp
Barbara Treutlein

Last updated by author(s): Jul 23, 2022

Reporting Summary

Nature Portfolio wishes to improve the reproducibility of the work that we publish. This form provides structure for consistency and transparency in reporting. For further information on Nature Portfolio policies, see our Editorial Policies and the Editorial Policy Checklist.

Statistics

For all statistical analyses, confirm that the following items are present in the figure legend, table legend, main text, or Methods section.

n/a	Confirmed	
☐	☒	The exact sample size (*n*) for each experimental group/condition, given as a discrete number and unit of measurement
☐	☒	A statement on whether measurements were taken from distinct samples or whether the same sample was measured repeatedly
☐	☒	The statistical test(s) used AND whether they are one- or two-sided<br>*Only common tests should be described solely by name; describe more complex techniques in the Methods section.*
☐	☒	A description of all covariates tested
☐	☒	A description of any assumptions or corrections, such as tests of normality and adjustment for multiple comparisons
☐	☒	A full description of the statistical parameters including central tendency (e.g. means) or other basic estimates (e.g. regression coefficient) AND variation (e.g. standard deviation) or associated estimates of uncertainty (e.g. confidence intervals)
☐	☒	For null hypothesis testing, the test statistic (e.g. *F*, *t*, *r*) with confidence intervals, effect sizes, degrees of freedom and *P* value noted<br>*Give P values as exact values whenever suitable.*
☒	☐	For Bayesian analysis, information on the choice of priors and Markov chain Monte Carlo settings
☒	☐	For hierarchical and complex designs, identification of the appropriate level for tests and full reporting of outcomes
☐	☒	Estimates of effect sizes (e.g. Cohen's *d*, Pearson's *r*), indicating how they were calculated

Our web collection on statistics for biologists contains articles on many of the points above.

Software and code

Policy information about availability of computer code

Data collection	See Methods. All codes are available upon request.
Data analysis	See Methods. The Pando R package is available on GitHub (https://github.com/quadbiolab/Pando). Other custom code used in the analyses is deposited on GitHub (https://github.com/quadbiolab/organoid_regulomes).

For manuscripts utilizing custom algorithms or software that are central to the research but not yet described in published literature, software must be made available to editors and reviewers. We strongly encourage code deposition in a community repository (e.g. GitHub). See the Nature Portfolio guidelines for submitting code & software for further information.

Data

Policy information about availability of data

All manuscripts must include a data availability statement. This statement should provide the following information, where applicable:

- Accession codes, unique identifiers, or web links for publicly available datasets
- A description of any restrictions on data availability
- For clinical datasets or third party data, please ensure that the statement adheres to our policy

Raw sequencing data will be deposited into ArrayExpress. Processed data and the VCF files for demultiplexing are deposited on Zenodo (https://doi.org/10.5281/zenodo.5242913).

Field-specific reporting

Please select the one below that is the best fit for your research. If you are not sure, read the appropriate sections before making your selection.

☒ Life sciences ☐ Behavioural & social sciences ☐ Ecological, evolutionary & environmental sciences

For a reference copy of the document with all sections, see nature.com/documents/nr-reporting-summary-flat.pdf

Life sciences study design

All studies must disclose on these points even when the disclosure is negative.

Sample size	Samples are listed in Supplementary Data 1.
Data exclusions	We excluded low quality and off-target cells using criteria as described in the Methods.
Replication	We analyzed multiple organoids from multiple cell lines for each different time point in the time course and multiple organoids for the perturbation experiment. To replicate the finding in the perturbation experiment we generated two different GLI3-KO lines and a control line in the same editing experiment and performed the multiome and sc-RNAseq experiment on both cell lines comparing to the generated control line. The loss of dorsal telencephalon and a strong enrichment in the ventral telencephalon was shown in two independent batches.
Randomization	Experiments were not randomized
Blinding	The organoids for the sc-RNAseq experiments of KO and WT organoids were performed in a blinded fashion. The organoids were chosen by a researcher not involved in the project. Investigators were not blinded during the other experiments.

Reporting for specific materials, systems and methods

We require information from authors about some types of materials, experimental systems and methods used in many studies. Here, indicate whether each material, system or method listed is relevant to your study. If you are not sure if a list item applies to your research, read the appropriate section before selecting a response.

Materials & experimental systems

n/a	Involved in the study	
☐	☒	Antibodies
☐	☒	Eukaryotic cell lines
☒	☐	Palaeontology and archaeology
☒	☐	Animals and other organisms
☒	☐	Human research participants
☒	☐	Clinical data
☒	☐	Dual use research of concern

Methods

n/a	Involved in the study	
☒	☐	ChIP-seq
☐	☒	Flow cytometry
☒	☐	MRI-based neuroimaging

Antibodies

Antibodies used	See Methods, in the sections "Westernblot", "Immunohistochemistry" and "Bulk Cut&Tag for GLI3 and H3K27ac"
Validation	See Methods, validations as provided by the manufacturer.

Eukaryotic cell lines

Policy information about cell lines

Cell line source(s)	See Methods, in the section "Stem cell and organoid culture".
Authentication	See Methods, in the section "Stem cell and organoid culture". Cells were further authenticated based on single cell RNA-Seq reads compared to single nucleotide polymorphisms.
Mycoplasma contamination	Cell lines were tested for mycoplasma contamination on a regular basis using a PCR-based test and were found to be negative for mycoplasma.
Commonly misidentified lines (See ICLAC register)	None.

Flow Cytometry

Plots

Confirm that:

- [x] The axis labels state the marker and fluorochrome used (e.g. CD4-FITC).
- [x] The axis scales are clearly visible. Include numbers along axes only for bottom left plot of group (a 'group' is an analysis of identical markers).
- [ ] All plots are contour plots with outliers or pseudocolor plots.
- [ ] A numerical value for number of cells or percentage (with statistics) is provided.

Methodology

Sample preparation	iPSC were brought into suspension with TrypLE and incubation of 3 min at RT. Cells were then resuspended in mTesr1 and spun down for 5 min 300g at 4 degrees. The pellet was resuspended in mTesR1 supplemented with 100 µg/ml Primocin and Y-27632 (final concentration 5µM). Cells were filtered trough a 30µm mesh prior to sorting. Cells were collected in mTesR1 supplemented with 100 µg/ml Primocin and Y-27632 (final concentration 5µM) and plated on matrigel. Organoids were dissocated with the papain-based dissociation kit (Miltenyi Biotec, 130-092-628) prior to sorting as described in Methods, filtered trough a 30µm mesh prior to sorting and cells were collected in PBS with 1% BSA.
Instrument	BD FACS Aria III and BD FACS Fusion
Software	FACS Diva
Cell population abundance	The final sorted population was around 11% of single cells for iPSC and 30% of single cells for two month organoid culture.
Gating strategy	See Extended Data Fig. 7c. Three gates were used 1) FSC-A vs SSC were used to gate for the bulk population of cells, 2) FSC-A vs FSH-H was used to minimize doublet sorting, 3) GFP+ populations were detemerined and gated by comparing to GFP negative controls.

- [x] Tick this box to confirm that a figure exemplifying the gating strategy is provided in the Supplementary Information.
